# Acetylation of conserved DVL-1 lysines regulates its nuclear translocation and binding to gene promoters in triple-negative breast cancer

**DOI:** 10.1038/s41598-019-52723-3

**Published:** 2019-11-07

**Authors:** Monica Sharma, Deborah Molehin, Isabel Castro-Piedras, Edgar G. Martinez, Kevin Pruitt

**Affiliations:** Department of Immunology and Molecular Microbiology, Texas Tech University Health Sciences Center, Lubbock, TX USA

**Keywords:** Breast cancer, Acetylation

## Abstract

Dishevelled (DVL) proteins are central mediators of the Wnt signalling pathway and are versatile regulators of several cellular processes, yet little is known about their post-translational regulation. Acetylation is a reversible post-translational modification (PTM) which regulates the function of several non-histone proteins involved in tumorigenesis. Since we previously demonstrated that lysine deacetylase, SIRT-1, regulates DVL protein levels and its function, we reasoned that DVL could potentially be a substrate for SIRT-1 mediated deacetylation. To further examine the potential role of multiple families of lysine deacetylases in the post-translational regulation of DVL, we screened for novel acetylation sites using liquid chromatography mass-spectrometry (LC-MS/MS) analysis. Herein, we report 12 DVL-1 lysine residues that show differential acetylation in response to changes in oxygen tension and deacetylase inhibition in triple-negative breast cancer (TNBC). PTMs are well documented to influence protein activity, and cellular localization. We also identify that acetylation of two key lysine residues, K69 and K285, present on the DIX and PDZ domains respectively, promote nuclear over cytoplasmic localization of DVL-1, and influences its promoter binding and regulation of genes implicated in cancer. Collectively, these findings for the first time, uncover acetylation as a novel layer of regulation of DVL-1 proteins.

## Introduction

The Wnt signalling pathway regulates fundamental aspects of growth and development, and its dysregulation promotes tumorigenesis^1,2^. Wnt ligands bind to their receptors and initiate intracellular signalling cascades that relay signals to two main branches: the canonical (Wnt/β-catenin) and the non-canonical (Wnt/PCP or Wnt/Ca^2+^) pathway. Canonical Wnt signalling promotes β-catenin stabilization in the cytoplasm and enables its translocation to the nucleus. Within the nucleus, β-catenin functions as a co-activator with lymphoid enhancer factor (LEF)/T-cell factor (TCF) transcription factors. Non-canonical Wnt signalling activates several β-catenin-independent pathways, which either lead to activation of planar cell polarity (PCP) or calcium pathways that influence cell motility^3^. Even though the Wnt pathway is widely studied, the mechanisms by which specific Wnt ligands signal into either the canonical and/or non-canonical pathway remains poorly understood^4^.

Dishevelled relays signals that arise from all Wnt ligands and it directs these signals to either canonical and/or non-canonical pathways to mediate cellular processes such as cell proliferation, migration, differentiation, polarity and stem cell renewal^5^. Structurally, DVL proteins consist of three highly conserved domains: an amino-terminal DIX domain, a central PDZ domain and a carboxyl-terminal DEP domain^3,6^. While the DIX domain aids DVL dimerization and formation of a signalosome, the PDZ and DEP domains facilitate interaction with Frizzled (FZD) and membrane localization^7^. Several studies show that these domains scaffold a variety of proteins and are essential for engaging different branches of the Wnt pathway^8,9^. DVL also harbours a nuclear export and a nuclear import signal^10^. Moreover, three isoforms of DVL (DVL1-3) are encoded by mammalian genes. Interestingly, even though DVL-1 is one of the least abundant of the total DVL protein pool, it has been shown to play an imperative role in stimulating LRP6 phosphorylation and activation of the Wnt transcriptional activity^11,12^. DVL is not only critical in human development^13^ and key physiological processes, but its altered expression has been demonstrated in some tumours relative to non-transformed cells and linked with various cancer types^3,14,15^. Even though DVL proteins bridge critical Wnt signals with nuclear responses, how it is regulated is poorly understood^16^.

Acetylation is an important PTM that affects histone^17–19^ and non-histone proteins, yet the role of non-histone acetylation is less clear^20^. Previously, we reported that SIRT-1 positively regulates DVL protein levels and in the present study further investigated this connection^21,22^. Here, for the first time, we report acetylation of 12 lysine residues present on conserved domains of DVL-1 protein, in response to oxygen tension and deacetylase inhibition in TNBC cells. Moreover, site-directed mutagenesis experiments reveal that acetylation of single lysine residues K69 (present on DIX domain) and K285 (present on PDZ domain) may potentially be acting as a regulatory switch for cytosolic to nuclear DVL-1 translocation. Our chromatin immunoprecipitation experiments revealed that K69 and K285 acetylation mimetics alter promoter binding of DVL-1 on tissue-specific promoters of *CYP19A1* gene which are deregulated in breast cancer^14,23^. Furthermore, we observed that acetylation-dependent DVL-1 promoter-binding also regulates I.4 and total aromatase transcript levels in TNBC cells. Therefore, this study is the first to reveal a novel mode of DVL regulation and reports acetylation as a novel driver of DVL-1 nuclear translocation and also suggests that acetylation may influence DVL’s role as a transcriptional regulator.

## Results

### **DVL-1 proteins are highly expressed in triple-negative breast cancer cells**

**Figure 1:**
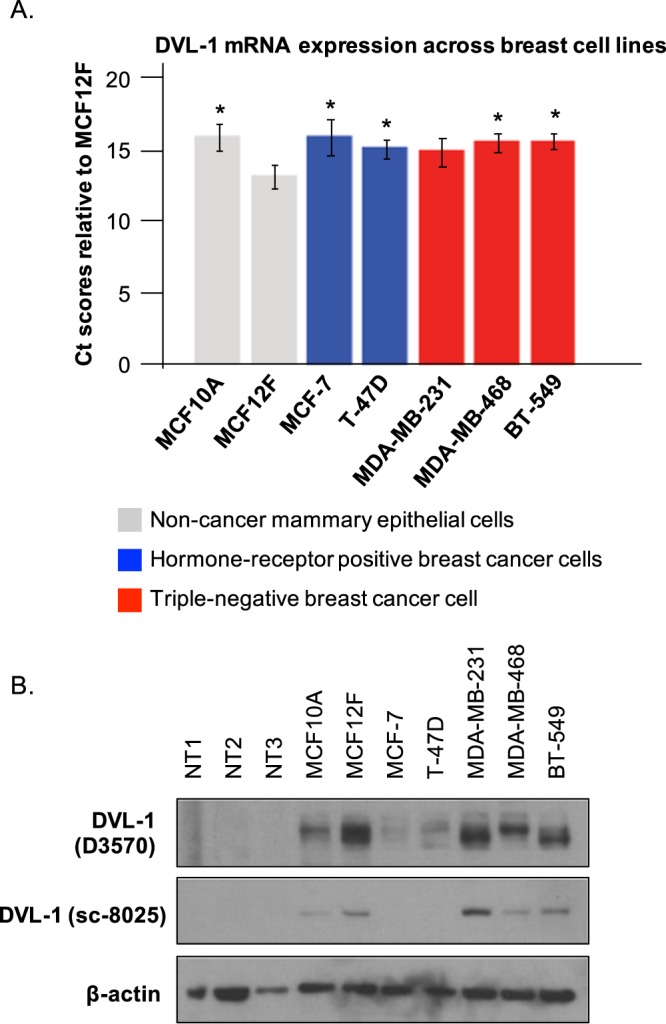
Dishevelled-1 proteins are highly expressed in triple-negative breast cancer cell lines. Total mRNA was isolated from different breast cancer cell lines: human non-cancer mammary epithelial cell line (MCF10A and MCF12F), hormone receptor positive breast cancer cells (MCF-7, T-47D) and triple-negative breast cancer cell lines (MDA-MB-231, BT-549 and MDA-MB-468). **(A)** Real-time PCR (qRT-PCR) analysis of endogenous *DVL-1* gene was performed using intron-spanning primers. All results are expressed as mean ± SEM and considered significant at *p < 0.05, **p < 0.01 and ***p < 0.001. **(B)** The protein expression patterns of endogenous DVL-1 were analysed by Western blotting in breast cells lines, as described above, along with breast normal whole tissue lysates (NT1, NT2, and NT3). The membranes were probed with two different DVL-1 specific antibodies (D3570; Sigma and sc-8025; Santa Cruz Biotechnology, Inc), and β-actin was included as a control (see Supplementary Fig. S1).

Because DVL-1 is implicated in tumorigenesis^15,24–26^ but remains poorly characterized, we analysed the relative mRNA and protein expression of DVL-1 in our panel of cancer cell lines. By performing real time quantitative polymerase chain reaction (qRT-PCR) across a panel of breast cancer cell lines and a non-cancer line using intron-spanning primers, we determined the mRNA expression of DVL-1. We found that DVL-1 mRNA levels did not vary considerably among the six cell lines (Figs 1A and S1A). Interestingly, however, we observed a more varied pattern of DVL-1 protein expression across the panel of cells lines screened using western blotting. We found that levels of DVL-1 proteins were relatively higher in triple-negative cells like MDA-MB-231, MDA-MB-468 and BT-549 cells compared to normal tissue lysates (NT) and hormone-receptor (ER/PR+) positive breast cancer cell lines (Figs 1B and S1B). Moreover, we observed high levels of DVL-1 proteins in immortal non-tumorigenic breast epithelial cell line, MCF12F, which are derived from a patient with fibrocystic breast disease that displayed focal areas of intraductal hyperplasia, a condition often associated with aberrant activation of Wnt signalling pathway^27^.

### **DVL-1 is acetylated at key lysine residues under different oxygen tension**

**Figure 2:**
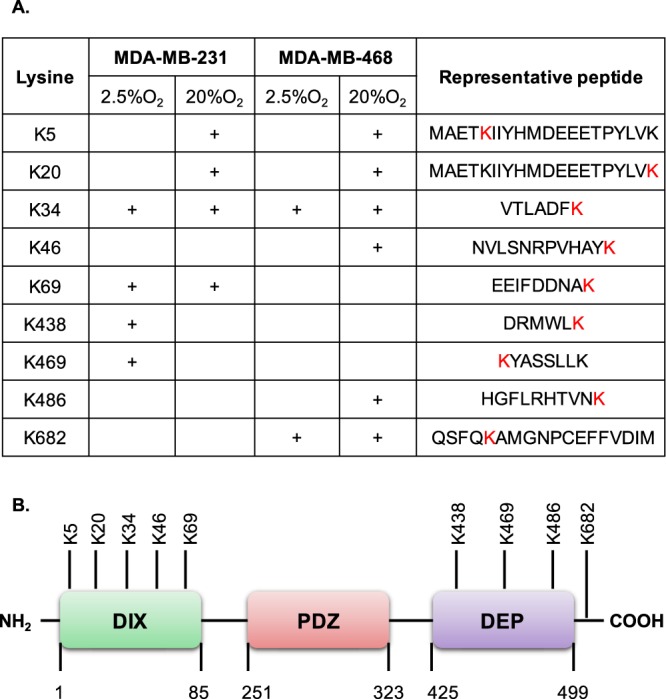
Putative lysine residues acetylated on endogenous DVL-1 under different oxygen tension and their approximate location on DVL structural domain. (**A)** The table indicates putative lysine residues on DVL-1 that were found to be acetylated under different oxygen tension (2.5% O_2_ and 20% O_2_) in MDA-MB-231 and MDA-MB-468 cells along with their representative peptide as detected by liquid chromatography mass spectrometry (LC-MS/MS) analyses. **(B)** Approximate representation of position of acetylated lysine (K) residues on DVL-1 conserved domain is shown. NH_2_ represents N-terminal; followed by amino-terminal DIX domain, a central PDZ, and a carboxyl-terminal DEP domain, and C representing C-terminal.

Nearly 50% of the advanced breast cancers exhibit low oxygen levels (≤2.5% O_2_, clinically termed as hypoxia) which directly or indirectly confer resistance to chemotherapeutic drugs leading to treatment failure^28–32^. Several studies have reported that hypoxic conditions alter Wnt/β-catenin signalling in order to meet the ever-changing needs of the tumor^33,34^. Moreover, exposure to low oxygen levels has been shown to regulate the activity of lysine modifying enzymes^35^. From our initial analysis, we found that the acetylation levels on endogenous DVL-1 proteins changed between two oxygen conditions (Fig. S2). Therefore, to determine whether oxygen tension influences DVL-1 acetylation patterns, we cultured cells at lower (2.5% O_2_) and atmospheric (20% O_2_) oxygen levels. To identify acetylation patterns on DVL-1 lysine residues under different oxygen levels, we performed immunoprecipitation with DVL-1 specific antibody followed by liquid chromatography mass spectrometry (LC-MS/MS). Interestingly, we identified nine novel acetylation sites on endogenous DVL-1 from LC-MS/MS analyses, which have not been previously identified. Remarkably, K34 was shown to be consistently acetylated under both oxygen conditions, suggesting that this PTM might be critical for DVL-1 function that is independent of oxygen tension. Moreover, acetylation on some lysine residues like K5, K20, K46, K438, K469, and K486 seemed to be sensitive to oxygen tension in MDA-MB-231 and MDA-MB-468 cells (Fig. 2A). Additionally, most of the acetylated lysine residues were concentrated in either the DIX or DEP domain (Fig. 2B). These results, for the first time, report that endogenous DVL-1 is acetylated and oxygen tension seems to influence its acetylation patterns in TNBC cell lines.

### **SIRT-1 and HDAC1 bind and deacetylate DVL-1 protein in multiple cell lines**

**Figure 3:**
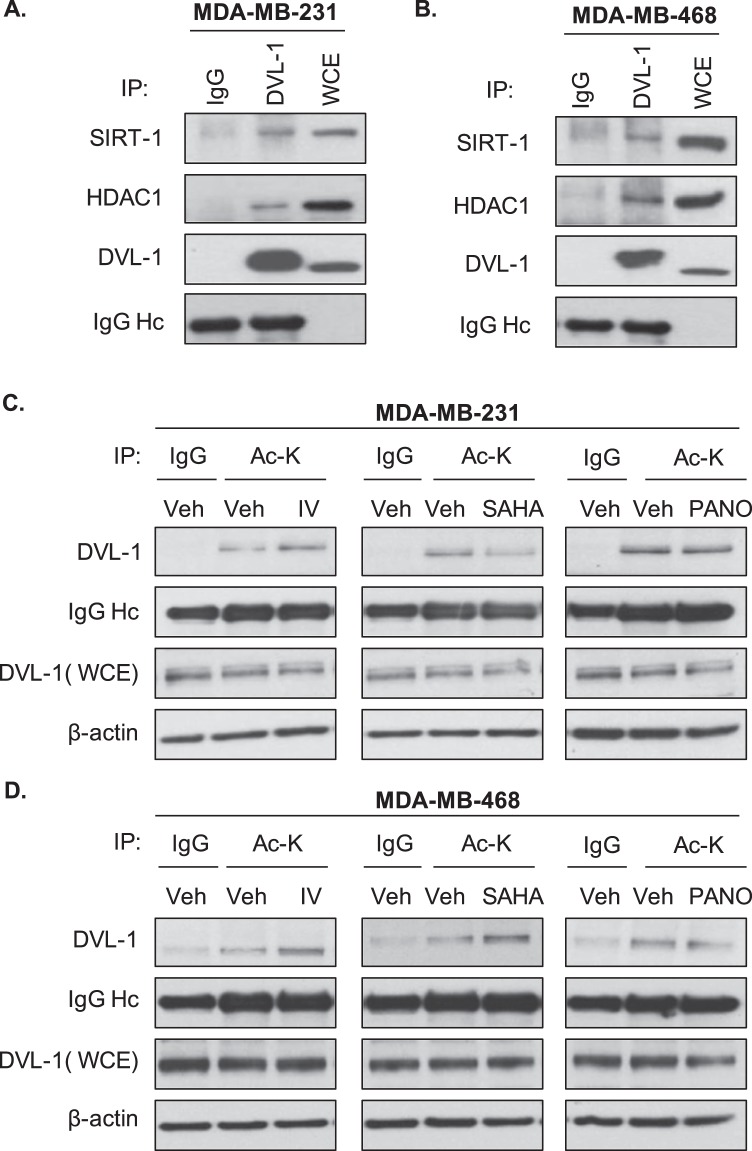
SIRT-1 and HDAC1 binds to DVL-1 protein and deacetylase inhibition alters DVL-1 acetylation levels in multiple cell lines. DVL-1 co-immunoprecipitates SIRT-1 and HDAC1 in **(A)** MDA-MB-231 and **(B)** MDA-MB-468 cells. Cells were used to extract protein lysates for immunoprecipitation (IP) of endogenous DVL-1, species-matched IgG was used as a negative control, and whole cell extracts (WCE) as a positive control. Western blotting was performed for SIRT-1 and HDAC1 (see Supplementary Fig. S4). Acetylation of DVL-1 is altered upon deacetylase inhibition compared to vehicle control in TNBC cells. **(C)** MDA-MB-231, and **(D)** MDA-MB-468 cells were treated with either DMSO (vehicle control; Veh) or 32 nM inhibitor-IV (IV), or 1 μM SAHA, or 6 nM Panobinostat (PANO) for 10 minutes. Equal amount of protein (2 μg) was loaded for each immunoprecipitation set up using acetyl-lysine (Ac-K) antibody as per protocol. Acetylation of DVL-1 was detected by western blotting along with positive control, whole cell extract (WCE) using DVL-1 specific antibody. Species-matched IgG was used as a negative control. IgG heavy chain (IgG Hc) was blotted for as a control for equal antibody loading for immunoprecipitation. β-actin was included as a loading control for WCE (see Supplementary Figs S5 and S6).

We also wanted to determine whether DVL-1 might be a novel deacetylation target of SIRT-1 given our previous reports^21,22^. Since we previously reported that SIRT-1 positively regulates DVL-1 function in MDA-MB-231 cells, we asked whether SIRT-1 binds DVL-1 in TNBC cells. We performed co-immunoprecipitation studies and found that DVL-1 interacts with SIRT-1. Extending analyses to other non-sirtuin deacetylases, we found that DVL-1 co-precipitates with HDAC1, a class I histone lysine deacetylase (HDAC), which is over-expressed in breast cancer cells (Fig. 3A). This observation was further corroborated in MDA-MB-468 cells, another TNBC cell line, which has high expression of DVL-1 proteins (Fig. 3B). We then reasoned that since DVL-1 interacts with SIRT-1 and HDAC1, it may be a substrate for lysine deacetylases.

Given the DVL-1 interaction with SIRT-1 and HDAC1, we wanted to determine whether inhibition of lysine deacetylases (KDACs) could possibly influence acetylation patterns on endogenous DVL-1. We treated two TNBC cells with 32 nM SIRT1-specific inhibitor IV (IV), an S-35 analog of EX-527; and FDA-approved pan-HDAC inhibitors, 1 μM Vorinostat (SAHA) and 6 nM Panobinostat (PANO) for 10 minutes (Fig. S3). MDA-MB-231 and MDA-MB-468 cells were included in this analysis since both these cell lines exhibit relatively higher DVL-1 protein expression. Following treatment with lysine deacetylase inhibitors (KDI), we performed immunoprecipitation using acetyl-lysine specific monoclonal antibodies followed by western blot for DVL-1. We found that SIRT-1 and pan-HDAC inhibitors altered DVL-1 acetylation patterns compared to vehicle control in MDA-MB-231 cells (Fig. 3C) as well as in MDA-MB-468 cells (Fig. 3D). Together, these results clearly demonstrate that DVL-1 is basally acetylated, a PTM which has never been reported on any isoform of DVL before. Moreover, our results are the first to demonstrate that SIRT-1 and HDACs regulate DVL-1 acetylation levels and pharmacological inhibition of these lysine deacetylases results in altered acetylation levels of DVL-1 in two triple-negative breast cancer cell lines.

### **Endogenous DVL-1 is acetylated at key lysine residues and KDI further influence its acetylation patterns**

**Figure 4:**
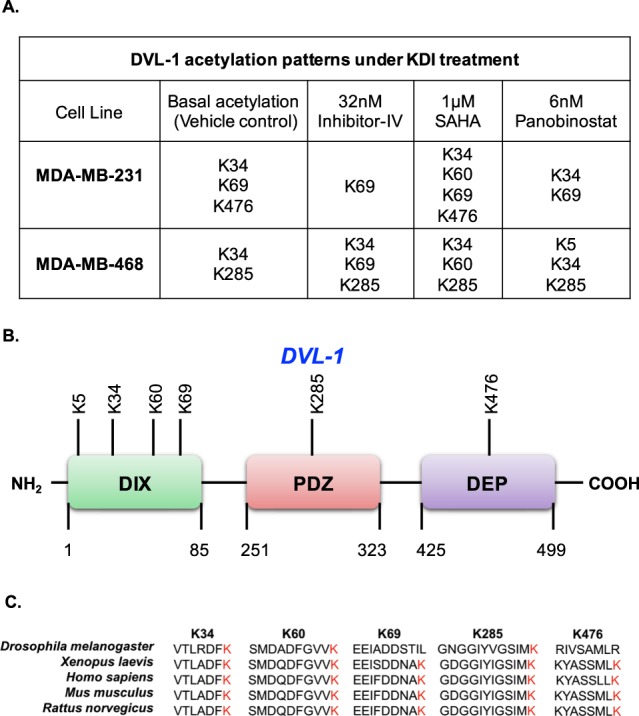
Endogenous DVL-1 is acetylated at key lysine residues. **(A)** The table indicates putative lysine residues that were found to be acetylated on DVL-1 under basal condition (vehicle control) and upon deacetylase inhibition (using Inhibitor-IV, SAHA and Panobinostat) and showed ion peaks at mass/charge (m/z) ratio of ∼126 in MDA-MB-231 and MDA-MB-468 cells. **(B)** Approximate representation of position of acetylated lysine (K) residues on DVL-1 conserved domain is shown. NH_2_ represents N-terminal; followed by amino-terminal DIX domain, a central PDZ, and a carboxyl-terminal DEP domain, and C representing C-terminal. **(C)** Multiple sequence alignment of DVL-1 acetylated peptide sequence (for K34, K60, K69, K285 and K476) across different organisms was performed using Clustal Omega software.

In order to further probe DVL-1 acetylation patterns, we began to systematically identify the specific lysine residues in endogenous DVL-1 that were acetylated upon lysine deacetylase inhibition. We inhibited SIRT-1, a class III lysine deacetylase, using 32 nM Inhibitor-IV, as well as the class I/II/IV lysine deacetylases using 1 μM SAHA and 6 nM panobinostat in MDA-MB-231 and MDA-MB-468 cells for 10 minutes. Next, we performed immunoprecipitation of endogenous DVL-1 (Fig. S7) and analysed the samples using LC-MS/MS. Figure 4A summarizes the putative lysine residues that were found to be acetylated and exhibited ion peaks at mass/charge (m/z) ratio of ~126 in MDA-MB-231 and MDA-MB-468 cells under basal (vehicle control) and KDI-induced conditions (i.e. cells treated with Inhibitor-IV, SAHA, and panobinostat) (also see Fig. S8A). In MDA-MB-231 cells, endogenous DVL-1 was acetylated at K34, K69, and K476 under basal conditions (i.e. vehicle control) with induction in acetylation on K60 upon treatment with 1 μM SAHA. Moreover, in MDA-MB-468 cells DVL-1 was acetylated on K34, and K285 under basal conditions (i.e. vehicle control) which was followed by a consistent increase in a distinct acetylation sites when cells were treated with different lysine deacetylase inhibitors. For instance, Inhibitor-IV induced acetylation on K69, SAHA induced K60 acetylation, and lastly, panobinostat resulted in induction in acetylation on K5 residue. We also wanted to determine DVL-1 acetylation status in a non-cancer setting, and therefore, we performed LC-MS/MS in MCF10A cells and surprisingly our results show that endogenous DVL-1 is acetylated in K34, K69, K438, and K476 in this cell line (Fig. S8B). These results indicate that acetylation PTMs might have an important regulatory role in DVL biology.

Taken together, these results indicate that DVL-1 is acetylated under basal and KDI-induced conditions. Interestingly, some of the lysine residues detected as acetylation sites such as K34, K60, K69, and K285 have been previously reported as sites which undergo ubiquitination in CYLD-deficient cells, where CYLD is a deubiquitinating enzyme which acts as a tumour suppressor gene^36^. More importantly, the acetylated lysine residues on endogenous DVL-1 protein were present on highly conserved domains i.e., at the N-terminus, in the DIX domain, in the central PDZ domain, and the C-terminus DEP domain; which are critical for DVL function^3^ (Fig. 4B). Of note, some of the lysine residues which were shown to be acetylated such as K34, K60, K69, K285, and K476 were highly conserved in DVL-1 orthologs from humans to Drosophila, suggesting that these residues may be critical for some evolutionary conserved functions of DVL-1 (Fig. 4C).

### **DVL-3 is acetylated on highly conserved DIX domain in TNBC cell lines**

**Figure 5:**
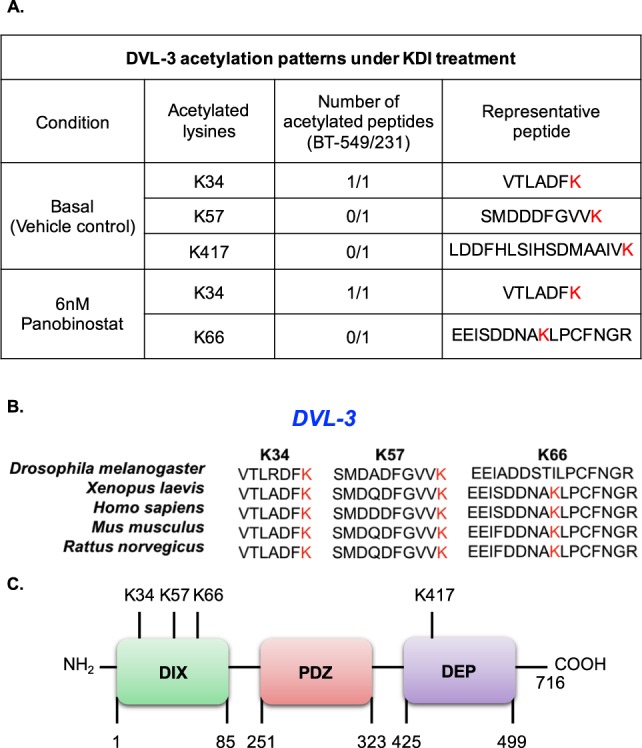
DVL-3 is acetylated on highly conserved DIX domain in MDA-MB-231 and BT-549 cells lines. (**A)** The table indicates putative lysine residues that were found to be acetylated on DVL-3 under basal and KDI-induced conditions in MDA-MB-231 and BT-549 cells along with their representative peptide as detected by liquid chromatography mass spectrometry (LC-MS/MS) analyses. **(B)** Multiple sequence alignment of DVL-3 acetylated peptide sequence (for K34, K57, and K66) across different organisms was performed using Clustal Omega software. **(C)** Approximate representation of position of acetylated lysine (K) residues on DVL-3 conserved domain is shown. NH_2_ represents N-terminal; followed by amino-terminal DIX domain, a central PDZ, and a carboxyl-terminal DEP domain, and C representing C-terminal.

To further determine whether other DVL proteins undergo acetylation, we mapped acetylation sites on DVL-3 in TNBC cells as well. We chose to analyse a third TNBC line, BT-549 cells which express DVL-3 along with MDA-MB-231 cells. We also focused on a single highly potent pan-HDAC inhibitor, Panobinostat, to access DVL-3 acetylation. BT-549 and MDA-MB-231 cells were treated with 6 nM panobinostat for 15 minutes followed by immunoprecipitation of DVL-3 and LC-MS/MS to identify acetylated lysine residues on DVL-3. Results from LC-MS/MS suggested that endogenous DVL-3 is acetylated on K34, K57, and K417 sites in vehicle control and panobinostat results in induction of acetylation on K66 in MDA-MB-231 cells. However, there was no alteration in acetylation patterns by panobinostat in BT-549 cells under these treatment conditions (Fig. 5A). Interestingly, most of the acetylated lysine residues on DVL-3 are present on the DIX domain, which is responsible for β-catenin dependent signalling, and is highly conserved in DVL-3 orthologs from humans to Drosophila (Fig. 5B,C). These data further demonstrate that certain lysine residues such as K34 on endogenous DVL proteins are subject to regulation by lysine deacetylases, and pharmacological inhibition of these KDACs influence acetylation patterns on highly conserved domains of DVL proteins.

### **Acetylation on K69 and K285 lysine residue alters subcellular localization of DVL-1 proteins in MDA-MB-231 and MDA-MB-468 cells**

**Figure 6:**
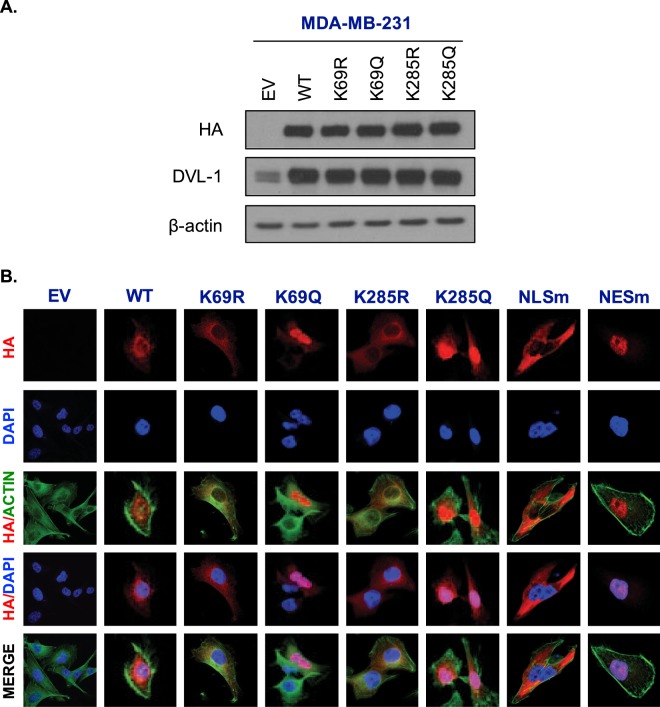
Acetylation on K69 and K285 lysine residue promotes DVL-1 nuclear localization in MDA-MB-231 cells. (**A)** Stable **e**xpression of empty vector (EV), N-terminal HA-epitope tagged DVL-1 wild type (WT), HA-tagged deacetylation mutants (K to R), HA-tagged acetylation mutants (K to Q) on two lysine residues, K69 and K285 in MDA-MB-231 cells. **(B)** Immunofluorescence staining of empty vector (EV), N-HA-tagged DVL-1 (WT), K69R mutant (N-HA-K69R), K69Q mutant (N-HA-K69Q), K285R mutant (N-HA-K285R), K285Q mutant (N-HA-K285Q), and controls such as a mutation on DVL-1 nuclear localization signal: N-HA-tagged DVL-1-NLS mutant (N-HA-NLSm), and a mutation on DVL-1 nuclear export signal: N-HA-tagged DVL-1-NES mutant (N-HA-NESm) proteins in stably expressing MDA-MB-231 cells. Merge of N-HA-DVL-1 (red) and nuclear staining (blue) proteins is shown as HA/DAPI for each of the mutant. Merge of actin (green) and N-HA-DVL-1 (red) proteins is shown as HA/Actin for each of the mutant.

**Figure 7:**
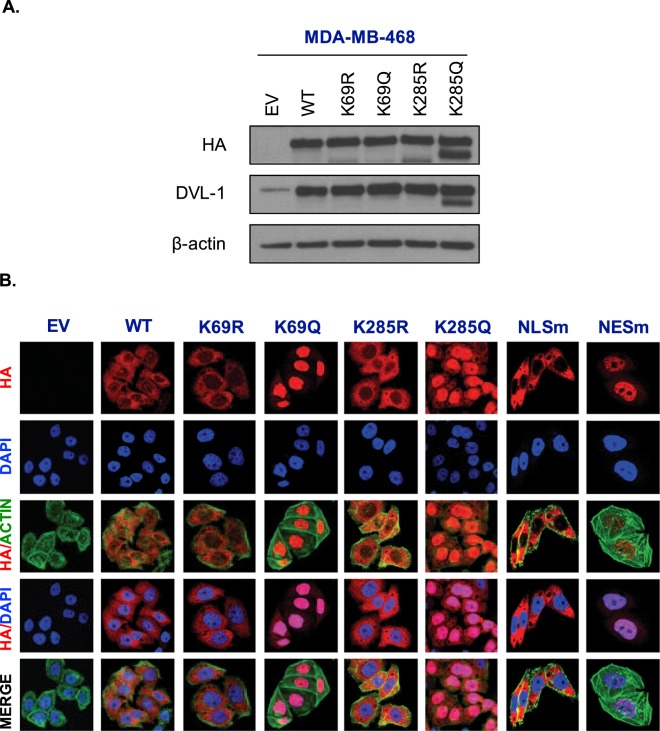
Acetylation on K69 and K285 lysine residue promotes DVL-1 nuclear localization in MDA-MB-468 cells. (**A)** Stable **e**xpression of empty vector (EV), N-terminal HA-epitope tagged DVL-1 wild type (WT), HA-tagged deacetylation mutants (K to R), HA-tagged acetylation mutants (K to Q) on two lysine residues, K69 and K285 in MDA-MB-468 cells. **(B)** Immunofluorescence staining of empty vector (EV), N-HA-tagged DVL-1 (WT), K69R mutant (N-HA-K69R), K69Q mutant (N-HA-K69Q), K285R mutant (N-HA-K285R), K285Q mutant (N-HA-K285Q), and controls such as a mutation on DVL-1 nuclear localization signal: N-HA-tagged DVL-1-NLS mutant (N-HA-NLSm), and a mutation on DVL-1 nuclear export signal: N-HA-tagged DVL-1-NES mutant (N-HA-NESm) proteins in stably expressing MDA-MB-468 cells. Merge of N-HA-DVL-1 (red) and nuclear staining (blue) proteins is shown as HA/DAPI for each of the mutant. Merge of actin (green) and N-HA-DVL-1 (red) proteins is shown as HA/Actin for each of the mutant.

Next we wanted to determine the impact of acetylation on DVL subcellular localization. In order to probe the functional significance of DVL-1 acetylation, we generated HA-tagged wild-type DVL-1 (full length N-HA-hDVL-1), HA-tagged deacetylation mimetics (K69R/K285R), HA-tagged acetylation mimetics (K69Q/K285Q). We also generated additional controls to rule out any potential side effect of mutation. These controls included HA-tagged-DVL-1 with a mutated nuclear localization signal (NLSm) and a mutated nuclear export signal (NESm). Once the mutations were confirmed by sequencing, we then transfected and selected MDA-MB 231 and MDA-MB-468 cells with the plasmids for stable expression. Since lysine acetylation neutralizes the positive charge, we chose arginine (R) and glutamine (Q) as a substitute because they are similar in length to lysine and R maintains its positive charge, mimicking deacetylation state, while Q is neutral, mimicking acetylation. Overexpression of N-HA-hDVL-1 constructs was confirmed by protein expression of WT and all four point mutants in MDA-MB-231 (Fig. 6A) and MDA-MB-468 cells (Fig. 7A).

Acetylation has been reported to change subcellular location of some proteins^20^. Here, using immunofluorescence assays, we detected that HA-tagged wild-type DVL-1 and deacetylation mutants (N-HA-hDVL-1 WT, K69R, and K285R) were mostly in the cytoplasm of stably expressing MDA-MB-231(Fig. 6B) and 468 cells (Fig. 7B). Interestingly, we observed that cells expressing K69Q and K285Q (acetylation mimetics) had strong nuclear staining. Moreover, the HA-tagged DVL-1 with the NLS-mutation (NLSm) and NES-mutation (NESm) localized largely to the cytoplasm and nucleus respectively. These controls further bolster the results with K69Q/K285Q and demonstrate that there was no side-effect with the process of mutation, selection or immunofluorescence in these cells. These results were reproduced in both MDA-MD-231 and MDA-MB-468 cells. In order to understand if acetylation on all or specific lysine residue influence DVL-1 cellular location, we created two more point mutants on another lysine residue on the DIX domain, K34R and K34Q. Interestingly, immunofluorescence staining in MDA-MB-231 cells stably expression K34R and K34Q mutants did not show any change in sub-cellular localization in comparison to K69Q and K285Q (Fig. S9A). These results demonstrate that acetylation of specific lysine residues, such as K69 and K285 but not K34, cause a switch in cellular localization of DVL-1 proteins in TNBC cells.

### K69 and K285 acetylation influences DVL-1 promoter binding and regulation of multiple *CYP19A1* tissue-specific promoters

**Figure 8:**
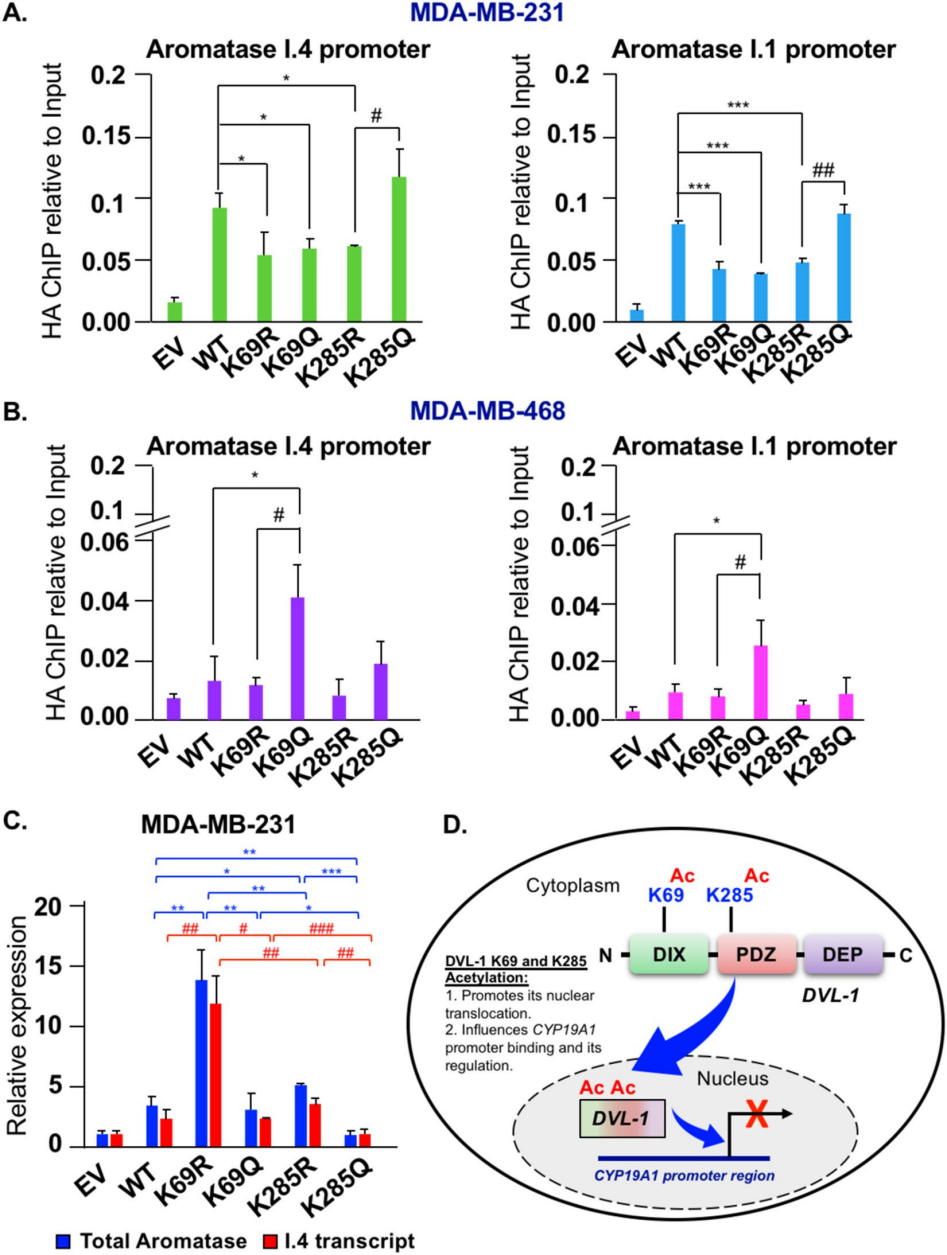
DVL-1 acetylation on K69 and K285 lysine residues influences its binding and regulation of *CYP19A1* (aromatase) tissue-specific promoters. Occupancy of HA-tagged DVL-1 at tissue-specific promoters of *CYP19A1* gene (I.4, and I.1) were analysed by real-time PCR. ChIP experiments for HA-tag were performed in **(A)** MDA-MB-231 and **(B)** MDA-MB-468 cells stably expressing EV (empty vector; negative control), HA-tagged wild-type DVL-1 (WT), HA-tagged deacetylation mutants (K69R and K285R), and HA-tagged acetylation mutants (K69Q and K285Q). The sign “*” represents significant change in HA-tagged DVL-1 binding between WT and mutants, while “#” represents significant change between R and Q mutants. **(C)** RNA was isolated from MDA-MB-231 cells stably expressing EV, WT, K69R/Q, and K285R/Q and cDNA was synthesized. Quantitative PCR was then performed using primers specific for adipose I.4 aromatase transcript (red), and the total aromatase (blue) mRNA with primers in the coding region common to all transcripts. **(D)** Schematic representation of the functional significance of acetylation on DVL-1 nuclear localization and its impact on binding and regulation of target genes such as *CYP19A1* promoter region. All results are expressed as mean ± SEM and considered significant at *^/#^p < 0.05, **^/##^p < 0.01 and ***^/###^p < 0.001.

After establishing that K69 and K285 acetylation promotes DVL-1 nuclear localization, we next wanted to determine whether acetylation-dependent nuclear translocation of DVL-1 also influences its promoter binding to genes implicated in cancer. We performed ChIP-quantitative real-time PCR (ChIP-qPCR) for HA-tag in MDA-MB-231 and MDA-MB-468 cells stably expressing different constructs such as empty vector (EV; pcDNA3.1 without HA-tag), HA-tagged full-length DVL-1 (wild type, WT), HA-tagged deacetylation mutants (K69R, K285R), as well as HA-tagged acetylation mutants (K69Q, K285Q). We previously demonstrated that DVL proteins bind multiple CYP19A1 tissue-specific promoters in multiple breast cancer cells including MDA-MB-231 and MDA-MB-468 cells^23^. CYP19A1 encodes for the aromatase protein and it is expressed in virtually every breast cancer line independent of ER expression status^23^. We also previously reported that DVL-1 was more linked with repression of specific aromatase transcripts while DVL-3 was more linked with activation. To further probe this novel link in MDA-MB-231 cells, we found that relative to WT, multiple mutants showed decreased binding to both adipose (I.4) and placental (I.1) promoter regions, while the K285Q showed increased binding relative to K285R at both promoters (Fig. 8A). In contrast, in MDA-MB-468 cells, we found that K69Q showed increased binding to both I.4 and I.1 relative to WT and K69R (Fig. 8B). Our results demonstrate that both acetylation and deacetylation mimetics alter DVL-1 binding to the placental (I.1) and adipose tissue (I.4) promoter regions of the aromatase gene with respect to WT. We further investigated whether differential binding of WT and mutants correlated with a change in I.4 and total aromatase transcript levels. We focused on the I.4 transcript since our previous findings suggested that DVL-1 binding to this tissue-specific promoter was more prominent. RNA was isolated from MDA-MB-231 cells stably expressing EV, WT, deacetylation (K69R, K285R) and acetylation (K69Q, K285Q) mutants. We used real-time qPCR with β-actin as an internal control to determine if WT versus mutants altered either the adipose (I.4) transcript or total aromatase mRNA. Interestingly, we found that with enrichment in promoter binding, K285 acetylation mimetic (K285Q) caused a statistically significant decrease in the I.4 and total aromatase transcripts with respect to WT (for total aromatase only) and deacetylation mutants (K285R, and K69R). Furthermore, we observed that with K69Q, there was a significant decrease in I.4 and total aromatase transcript with respect to K69R. In contrast, deacetylation state of K69 and K285 (R) caused a significant increase in the levels of I.4 and total aromatase transcript (Fig. 8C). These results are showing the general trend that increased DVL-1 promoter occupancy correlates with decreased aromatase transcripts is consistent with our previous report that DVL-1 was more linked with repression of aromatase. Together, these data demonstrate that post-translational acetylation serves as regulatory switch for DVL-1 proteins. Not only does acetylation play an important role in DVL-1 nuclear translocation, but also appears to influence its binding to multiple aromatase promoters, as well as differentially regulates tissue-specific aromatase transcripts that are aberrantly activated in cancer (Fig. 8D).

## Discussion

DVL plays a key role in integrating and transmitting Wnt signals regardless of whether they are normal or aberrant in nature. However, how DVL relays complex signals from upstream receptors to downstream signalling components and how it conducts this symphony of activity remains poorly understood. Our findings here provide additional insight into DVL post-translational regulation beyond the previously described PTMs such as phosphorylation, methylation, and ubiquitination^3,5,37^. Here, for the first time, we report as well as demonstrate functional significance of acetylation as a novel post-translational modification on DVL family members, in response to oxygen tension and deacetylase inhibition in triple-negative breast cancer cells.

The Wnt signalling pathway plays a pivotal role in embryonic development and homeostasis. It is well established that deregulation of this pathway contributes to the initiation and progression of human diseases such as cancer by upregulation of genes which directly control major cellular phenotypes such as proliferation, migration, survival and matrix remodeling^38^. Interestingly, the over-active Wnt signalling pathway is considered a potential therapeutic target in triple-negative breast cancer, a highly aggressive subtype of breast cancer with elevated metastatic potential and invasiveness^39–42^. Moreover, recent studies demonstrate that TNBC cells acquire metastatic-associated phenotypes via upregulation of Wnt pathway components, including DVL-1^43^. Several reports have demonstrated elevated levels of DVL-1 proteins compared to normal counterparts and its role in tumorigenesis^14,15^. It has been demonstrated that over-expression of DVL is essential for β-catenin/TCF-dependent transcriptional activation, cell proliferation in cancer settings^26,44–46^ and is associated with poor prognosis and lower survival rate in lung cancer^25^. Furthermore, down-regulation of DVL proteins were shown to inhibit cell proliferation and invasion in hepatoblastoma^44^. Recently over-expression of DVL 1–3 were shown to induce expression of ATP-binding cassette (ABC) superfamily of membrane transporters which resulted in multi-drug-resistance in colorectal cancer cell lines^24^. Despite the clear importance of DVL-1 in normal and pathophysiological settings, much remains unknown about how it is regulated. Herein, we demonstrate that DVL-1 proteins are over-expressed in TNBC cells compared to normal tissue lysates (NT) and hormone-receptor (ER/PR+) positive breast cancer cell lines. Interestingly, the mRNA levels of DVL-1 were relatively similar, however, the protein levels differed between the cell lines, further suggesting the critical nature of post-translational control.

Several studies have reported that hypoxic conditions alter Wnt/β-catenin signalling in order to meet the ever-changing needs of the tumor^33,34,47^. Moreover, exposure to low oxygen levels has been shown to regulate the activity of acetyltransferase, deacetylase and sirtuin activity^35^. Based on other seminal reports that oxygen regulates Wnt signalling pathway and the activity of these acetylation-modifying enzymes, we asked whether oxygen tension influences acetylation patterns on DVL-1 proteins. This study, for the first time demonstrates nine novel acetylation sites on highly conserved domains of endogenous DVL-1 proteins under different oxygen tension. To note, some of the lysine residues, such as K34, were acetylated in multiple cell lines, independent of the oxygen levels, indicating that it might be critical for regulating DVL-1 protein function which has yet to be discovered.

Our study also focuses on mapping KDI-induced DVL-1 and DVL-3 acetylation sites in TNBC. We previously reported that SIRT-1, a class III lysine deacetylase, positively regulates DVL-1 proteins levels in cancer cells^21^. Moreover, we also reported that SIRT-1 inhibition, using a SIRT-1 specific Inhibitor-IV (IV), resulted in a significant decrease in DVL-1 binding with TIAM1 in MDA-MB-231 cells^22^. However, this study examined more broadly the inhibition of SIRT-1 and class I/II/IV deacetylases in the regulation of acetylation patterns on endogenous DVL proteins across multiple TNBC cell lines. It has been reported that aberrant lysine acetylation of non-histone proteins can influence tumour progression and metastasis^48^. Historically, deacetylases have shown to regulate epigenetic modifications. However, there has been a steady increase in the number of non-histone proteins identified as acetylation targets. This uncovers another layer of regulation of DVL at the post-translational level and further shows that KDIs may elicit their effect in part through previously unidentified non-histone target proteins such as DVLs.

Recent studies report KDI therapy as an effective therapy for treatment of chemo-resistant TNBC cells. There has been an emerging interest in using FDA-approved KDI, as novel targeted therapies for TNBC patients. One early study investigated Vorinostat (also known as SAHA) for anti-cancer therapy and its ability to impact tumour growth. The report suggested that Vorinostat suppressed cancer cell proliferation by inducing cell-cycle arrest and/or apoptosis and reduced angiogenesis^49^. Similarly, Panobinostat, reduced cell migration and invasive potential of TNBC cells at low nanomolar concentrations^50^ selectively targeting TNBC cell proliferation and survival *in vitro* and tumorigenesis *in vivo*, respectively^51–53^. The present study uncovers a new link between the activity of deacetylases and DVL proteins. The findings described here have generated the very first map of six acetylation sites in response to KDAC-mediated deacetylation of endogenous DVL-1 proteins, which are present on highly conserved functional domains of DVL. Interestingly, the acetylated lysine residues reported in this study, are situated in domains which are very critical for DVL function. For instance, there is a heavy deposition of acetylation sites on lysines (K5, K20, K34, K46, K57, K60, K66, K69) which are a part of highly conserved DIX domain, a signalosome-initiating domain responsible for relaying signals into Wnt/β-catenin dependent pathway. Interestingly, the acetylated lysines in the DIX domain are also targets for other modifications such as ubiquitination^54,55^. Moreover, we mapped acetylation on lysine residues which belonged to the PDZ (K285) and DEP (K417, K438, K469, K476, K486 and K682) domain which are responsible for interaction with Fzd receptors, and for DVL membrane localization, respectively. Of note, some of the lysines (K34, K57, K60, K66, K69, K285, and K476) were found to be highly conserved on DVL-1 and DVL-3 across different species analysed, suggesting that these lysine residues play a crucial role in the DVL function. Importantly, two acetylated lysines identified in our study (K60 and K69) have already been reported to play a critical role in regulating DVL function and would likely alter Wnt signalling^56,57^.

Acetylation has been shown to be a key PTM involved in regulating metabolic enzymes activity^58^, protein-protein and protein-DNA interaction and sub-cellular localization^20,59^. The present study reports acetylation as a potential regulatory switch which regulates DVL-1 translocation into the nucleus of TNBC cells. Previous studies have elegantly demonstrated that DVL nuclear localization is required for canonical Wnt signaling^10,60^. However, the mechanistic basis for this remains unclear^16^. Our results for the first time indicate that acetylation of single lysines such as K69 (located on the DIX domain) and K285 (located on the PDZ domain) promotes DVL-1 translocation into the nucleus. Interestingly, these sites also regulate DVL-1 promoter binding to tissue-specific aromatase promoters. Furthermore, our results show that acetylation-driven promoter binding of DVL-1 also results in differential regulation of tissue-specific aromatase transcript levels.

In summary, this report examines DVL biology and demonstrates its novel link with deacetylases in pathophysiological settings. The present report focuses on DVL-1 and DVL-3 protein as novel deacetylase substrates, which uncovers a new regulatory dimension to DVL biology. We now know that DVL-1 and DVL-3 proteins, are post-translationally regulated at yet another level and treatment with specific KDIs may impact their acetylation patterns on highly conserved domains. Furthermore, we also demonstrate that acetylation of specific lysine, K69 and K285 may potentially act as regulatory switch for nuclear translocation and promoter occupancy of DVL-1 proteins. Taken together, this report provides new insight into an important but poorly understood process of post-translational regulation of DVL. Our findings are particularly relevant within the context of TNBC, a cancer subtype that lacks targeted treatment options, which could help identify novel therapeutic vulnerabilities.

## Methods

### Cell culture

All cell lines (MCF7, T47D, MDA-MB-231, MDA-MB-468, BT-549, MCF10A and MCF12F) used in this manuscript were purchased from ATCC which utilizes STR technology for Cell Authentication, and they were used in a low passage (<20) within 6 months or less after receipt or resuscitation. MB231 cells were cultured in DMEM (Gibco) supplemented with Na Pyruvate (Sigma), MCF7 cells were cultured in MEM (Gibco) supplemented with 0.1% insulin (Sigma), MCF10A and MCF12F cells were cultured in HuMEC Medium (Gibco) supplemented with HuMEC supplement kit (Gibco); while T47D, BT549 and MB468 cells were cultured in RPMI 1640 (Gibco) supplemented with 0.1% insulin. All culture media were supplemented with 10% fetal bovine serum and 1% penicillin/streptomycin (Invitrogen).

### mRNA expression analysis

Total RNA was isolated from cells using a Pure-link RNA mini kit (Invitrogen). 2 μg of total RNA was reverse-transcribed using SuperScriptIII Reverse Transcriptase (ThermoFisher) to synthesize first-strand of complementary DNA (cDNA). Intron-spanning primers were designed for each specific target DNA and gene expression measured by endpoint-PCR using JumpStart RedTaq (Sigma). The primers listed in Table 1 were used for expression analysis for Fig. 1A and 8C.

**Table 1:** Real time qPCR primers used for the study.

	Forward Primer	Reverse primer
DVL-1	5′-GGGAGTCAGCAGAGTGAAGG-3′	5′-CACCACTGTATAGGCCTTGGTC-3′
I.4 promoter	5′-GAGGTCACAGAAGGCAGAGG-3′	5′-GAGGGGGCAATTTAGAGTCC-3′
Total aromatase	5′-GGGATCGGCAGTGCCTGCAA-3′	5′-AACAAGGCCGGGGCCTGACA-3′
Beta actin	5′-GGACTTCGAGCAAGAGATGG-3′	5′-AGCACTGTGTTGGCGTACAG-3′

### End-point PCR and quantitative real-time qRT-PCR

End-point PCR amplification was performed using JumpStart RedTaq (Sigma). The Applied Biosystems Veriti 96-well thermal cycler (Applied Biosystems) and Gel DOC EZ imager (Bio-Rad) were used analyses. Gene expression was quantified by real-time qPCR in CFX96 Deep-Well Real-Time System (BioRad) using PerfeCta SYBR Green FastMix and specific oligonucleotide primers. The reaction mixtures contained 10 µl PerfeCta SYBR Green FastMix, 7.2 µl ddH2O, 2.0 µl template cDNA and 0.4 µl gene-specific 10 µM PCR oligonucleotides primers. The reaction conditions were 95 °C for 30 s, followed by 40 cycles of 95 °C for 5 s and 60 °C for 30 s and Melt Curve (dissociation stage). Relative gene expression was calculated as delta (∆ Re (the difference between the cycle threshold values, Ct, of the internal control, and Ct of gene of interest) and confirmed by 2–∆∆ CT method.

### Normal tissue lysates

Human breast whole tissue lysates (normal) were purchased from GeneTex (GTX24006), Novus Biological (NB820-59203) and G-Biosciences (NLH-13). Total proteins were extracted in RIPA buffer along with protease inhibitor cocktail.

### Western blots

Samples were subjected to polyacrylamide gel electrophoresis using Invitrogen Bolt gel system, transferred to PVDF (Millipore) membranes, and immunoblotted. Antibodies used are as follows: DVL-1 (D3570; Sigma and sc-8025; Santa Cruz), SIRT-1 (DB083; Santa Cruz), HDAC1 (sc-7872; Santa Cruz), HA (cst-3724; Cell Signalling) and β-actin (sc-47778; Santa Cruz). Membranes were incubated in 5%milk/TBST with primary antibody overnight at 4 °C. Membranes were washed with TBST and probed with horseradish peroxidase-conjugated secondary antibodies in 5%milk/TBST for 1 hr/room temperature (RT). Membranes were visualization by enhanced chemiluminescence (ECL) reagent (Thermo Scientific) on premium X-ray films (Phenix Research).

### Co-immunoprecipitation

Cells were seeded and incubated at 37 °C under conventional O_2_ conditions. Once they were 70% confluent, cells were washed with PBS and lysed in Co-IP lysis buffer (50 mM Tris-HCl pH 7.5, 100 mM NaCl, 1% NP-40, 10% Glycerol, 1 mM DTT, 0.5 mM EDTA, 0.5 mM EGTA, 0.5 mM NaF and Protease inhibitor cocktail) or in a much gentle lysis buffer (25 mM Tris, pH 7.4,150 mM NaCl, 1 mM EDTA, 1% NP-40, 5% glycerol and Protease inhibitor cocktail). This was followed by quantification for equal protein loading using standard BCA protocol (Thermo Scientific). Cell extracts were incubated with 2 μg of DVL-1 specific antibody (D3570; Sigma), and species-matched IgG as negative control for 2 hrs/4 °C. Protein A Dynabeads (Life Technologies) were incubated with the antigen-antibody complex for 2 hrs/4 °C. Beads were washed four times with lysis buffer with gentle agitation for 5 mins per wash. 5x sample buffer (Invitrogen) was used for elution of complex from beads followed by Western blotting along with WCE.

### Immunoprecipitation

Cells were treated with DMSO, 32 nM inhibitor-IV, 1 μM SAHA, 6 nM Panobinostat for 10 mins and harvested in acetylation lysis buffer (acKIP) (1% Triton X-100, 0.5% NP-40, 150 mM NaCl, 50 mM Tris (pH 7.4), and 10% glycerol, with complete protease inhibitor cocktail, 1 µM TrichostatinA and 1 mM nicotinamide). Protein concentration was quantified by the BCA method and 2 mg protein from each sample were prepared for immunoprecipitation (IP) in a 1 ml volume of acKIP buffer. IP was performed using 2 μg anti-acetyl lysine antibody (AAC01; Cytoskeletal) and species-matched mouse IgG for 2 hrs/4 °C. Protein G Dynabeads (Invitrogen) were added to the immune-complex and incubated for 2 hrs/4 °C. Beads were washed and samples were eluted in the same way as Co-IP protocol.

### Liquid chromatography/mass spectrometry (LC-MS/MS)

Cells were cultured and seeded at 37 °C under lower (2.5%O_2_) and atmospheric (20%O_2_) oxygen conditions. Once 70% confluent, cells were washed with PBS and harvested in RIPA buffer. Protein extracts were incubated with 4 μg of anti-DVL-1 antibody (D3570; Sigma) or anti-DVL-3 antibody (SAB4200007; Sigma) for 2 hrs/4 °C. Protein A dynabeads (Invitrogen) were added to the immune-complex and incubated for 2 hrs/4 °C. IP protocol was followed as mentioned above. Dry beads were shipped to Applied Biomics Inc. (Hayward, CA) for acetylation site identification by LC-MS/MS mass spectrometry.

### HA-hDVL-1 pcDNA3.1 (+) construct generation

The full-length 2.1-kb DVL-1 gene was cloned into KpnI/EcoRI restriction enzyme sites of 5.4-kb pcDNA3.1(+) mammalian expression plasmid using T4 DNA ligase (Invitrogen). Synthetic nucleotide primers (Table 2) used were designed with HA-epitope tag at the amino-terminus. MAX efficiency competent cells (DH5-alpha; Thermofisher) were transformed with ligation mixture, and plated on LB-ampicillin agar at 37 °C overnight. Bacteria colonies were screened and plasmids extracted by the QIAprep spin miniprep Kit (Qiagen). Plasmids isolated were subjected to KpnI and EcoRI restriction digest, and fragments were resolved on 1% agarose gel (Bio-Rad) in 1X TAE buffer. Sanger plasmid sequencing was used to confirm the DNA sequences.

**Table 2:** pcDNA3.1 (+) – HA tag hDVL-1 PRIMER SEQUENCE (at N terminus).

Primers	Sequence (5′ to 3′)
N-HA-hDVL-1 F primer	5′- tt aag ctt ggt acc atg tac cca tac gat gtt cca gat tac gct gcg gag acc aag att atc tac cac -3′
N-HA-hDVL-1 R primer	5′-gga tat ctg ca gaa ttc tca cat gat gtc cac gaa gaa ctc gca ggg gtt ccc-3′

### Site-directed mutagenesis

pcDNA3.1-N-HA-hDVL-1 plasmids were subjected to site-directed mutagenesis with nucleotide primers (Table 3) designed using the QuickChangeII-XL site-directed mutagenesis primer design tool and kit (Agilent Technologies) following the manufacturer’s protocol. Plasmids were transformed into XL10-Gold ultra-competent cells and plated on ampicillin impregnated LB-agar overnight at 37 °C. Bacteria colonies were screened and plasmids purified using Qiagen miniprep kit. Sanger plasmid sequencing was done to validate the mutations.

**Table 3:** Site-directed primers for DVL-1 acetylation sites.

Site	Forward Primer	Reverse primer
K34R	5′-tgctgagcacgttcctgaagtcggccagc-3′	5′-gctggccgacttcaggaacgtgctcagca-3′
K34Q	5′-gctgagcacgttctggaagtcggccagcg-3′	5′-cgctggccgacttccagaacgtgctcagc-3′
K69R	5′-aagcagggaagcctggcattgtcatcaaagatctc-3′	5′-gagatctttgatgacaatgccaggcttccctgctt-3′
K69Q	5′-gttgaagcagggaagctgggcattgtcatcaaaga-3′	5′-tctttgatgacaatgcccagcttccctgcttcaac-3′
K285R	5′-gccccgcccctcatgatggagccaatg-3′	5′-cattggctccatcatgaggggcggggc-3′
K285Q	5′-cagccccgccctgcatgatggagccaatgt-3′	5′-acattggctccatcatgcagggcggggctg-3′
NLSm	5′-cagcacttggccacagcgccgctggcgggccccgtctgggaa-3′	5′-ttcccagacggggcccgccagcggcgctgtggccaagtgctg-3′
NESm	5′-cactggagccactgttggcgttcgcggtggcgagattgctgc-3′	5′-gcagcaatctcgccaccgcgaacgccaacagtggctccagtg-3′

### Stable expression of WT and mutants in MDA-MB-231 and 468 cell lines

350,000cells were plated in a p6-well dish. After 24 hrs, cells were transfected with 1 μg DNA empty vector (pcDNA3.1(+) without HA-tag), full-length DVL-1 (WT), K to R, K to Q, NLS and NES point-mutants using lipofectamine3000 reagent (Thermo Scientific). After 24 hrs, the cells were selected using 1 mg/ml G418 antibiotic until no cells were remaining in non-transfected control.

### Immunofluorescence

3.5 × 10^5^ cells were seeded onto coverslips (12 mm) in a 60 mm tissue culture dish. The cells were fixed with 4% paraformaldehyde for 15 mins/RT, followed by quenching with 50 mM ammonium chloride (NH_4_Cl), permeabilization with 1% Triton X-100 for 10 mins. The coverslips were blocked with 5%BSA for 30 mins, followed by 1 hr incubation with the primary antibody, HA (cst-3724; Cell Signalling). Samples were then incubated with secondary antibodies: Alexa flour 568 #A11036, and phalloidin 488 #A12379 (Thermo Scientific) for 1 hr/RT. The samples were rinsed in PBS for 5 mins and mounted with prolong gold antifade mounting solution with DAPI (P36941, Thermo Scientific), then cured overnight at RT and stored at −20 °C until imaged. The samples were imaged using a laser scanning confocal microscope Nikon T-1E with a 60x objective and NIS software.

### Chromatin immunoprecipitation

Cells were grown to confluence in 100 mm dishes; a final count of approximately 5 × 10^6^cells per plate. Proteins were cross-linked to DNA using formaldehyde (Sigma) added directly to the culture medium at a final concentration of 1% for 8 mins/RT. The cross-linking reaction was quenched by adding glycine (Sigma) to a final concentration of 0.125 M for 5 mins/RT. The medium was removed, and cells were washed twice with PBS. Cells were scraped, pelleted and washed twice with PBS plus protease inhibitor cocktail. Cells were resuspended in SDS Lysis buffer (50 mM Tris-HCl pH 8.0, 10 mM 0.5 M EDTA, and 1% SDS) with protease inhibitor cocktail. MDA-MB-231 and MDA-MB-468 cells were sonicated in a Diagenode Bioruptor sonicator for 25 and 20 cycles (30 sec pulses and 30 sec rest) respectively. The soluble chromatin fraction was quantitated and 100 μg of chromatin was incubated for 2 hrs/4 °C with HA (cst-3724; Cell Signalling). Next, 11 μl of Dynabeads Protein A (Invitrogen) was added to the chromatin-antibody mixture and incubated with rotation for 2 hrs/4 °C. ChIPs were washed with five low salt wash buffer (0.1% SDS, 1% Triton X-100, 2 mM EDTA, 20 mM Tris HCl pH 8.1, and 150 mM NaCl), three high salt wash buffer (0.1% SDS, 1% Triton X-100, 2 mM EDTA, 20 mM Tris HCl pH 8.1, and 500 mM NaCl), and one TE wash (1 mM EDTA and 10 mM Tris HCl pH 8). Crosslinks were reversed overnight at 65 °C, followed by RNAse (Promega) at 37 °C/2 hrs, and proteinase K incubation (Promega) at 55 °C/2 hrs. DNA was eluted using Qiaquick PCR purification kit (Qiagen) and amplified by end-point PCR and real time qPCR using gene-specific primers (Table 4).

**Table 4:** ChIP primers used for the study.

Promoter region	Forward primer (5′ to 3′)	Reverse primer (5′ to 3′)
Aromatase I.4	CACTCACCTGGCACCTAACC	AAGAGCCACACACTGGGAAG
Aromatase I.1	TCACCCCCAACACATAGCAC	CCACCACACACCACATTGTTC

### Statistical analysis

Statistical analysis was performed using unpaired Student’s t-test to assess whether differences observed in the various experiments were significant. All results are expressed as mean ± SEM and considered significant at ^*/#^p < 0.05, ^**/##^p < 0.01 and ^***/###^p < 0.001.

### *In silico* analysis

Multiple sequence alignment of DVL-1 and DVL-3 across different organisms was performed using Clustal Omega software (https://www.ebi.ac.uk/Tools/msa/clustalo/).

## Supplementary information

Supplementary Figures
