# Supplementary material for: Acetylation of conserved DVL-1 lysines regulates its nuclear translocation and binding to gene promoters in triple-negative breast cancer
Source: Sci Rep. 2019 Nov 7;9:16257. doi: 10.1038/s41598-019-52723-3 (PMC6838061; doi:10.1038/s41598-019-52723-3)
Supplement: Supplementary file 1 — Supplementary Figures [file 41598_2019_52723_MOESM1_ESM.pdf]

Acetylation of conserved DVL-1 lysines regulates its nuclear translocation and binding to gene promoters in triple-negative breast cancer

Monica Sharma, Deborah Molehin, Isabel Castro Piedras, Edgar Martinez, and Kevin Pruitt*

Department of Immunology and Molecular Microbiology, Texas Tech University Health Sciences Center, Lubbock, TX, USA

*Address correspondence to: Kevin Pruitt, Ph.D., Department of Immunology & Molecular Microbiology, Texas Tech University Health Sciences Center, 3601 4th Street, Lubbock, Texas 79430-6591, (ph) 806-743-2523; (fax) 806-743-2334, Email: kevin.pruitt@ttuhsc.edu

Supplementary Figure S1

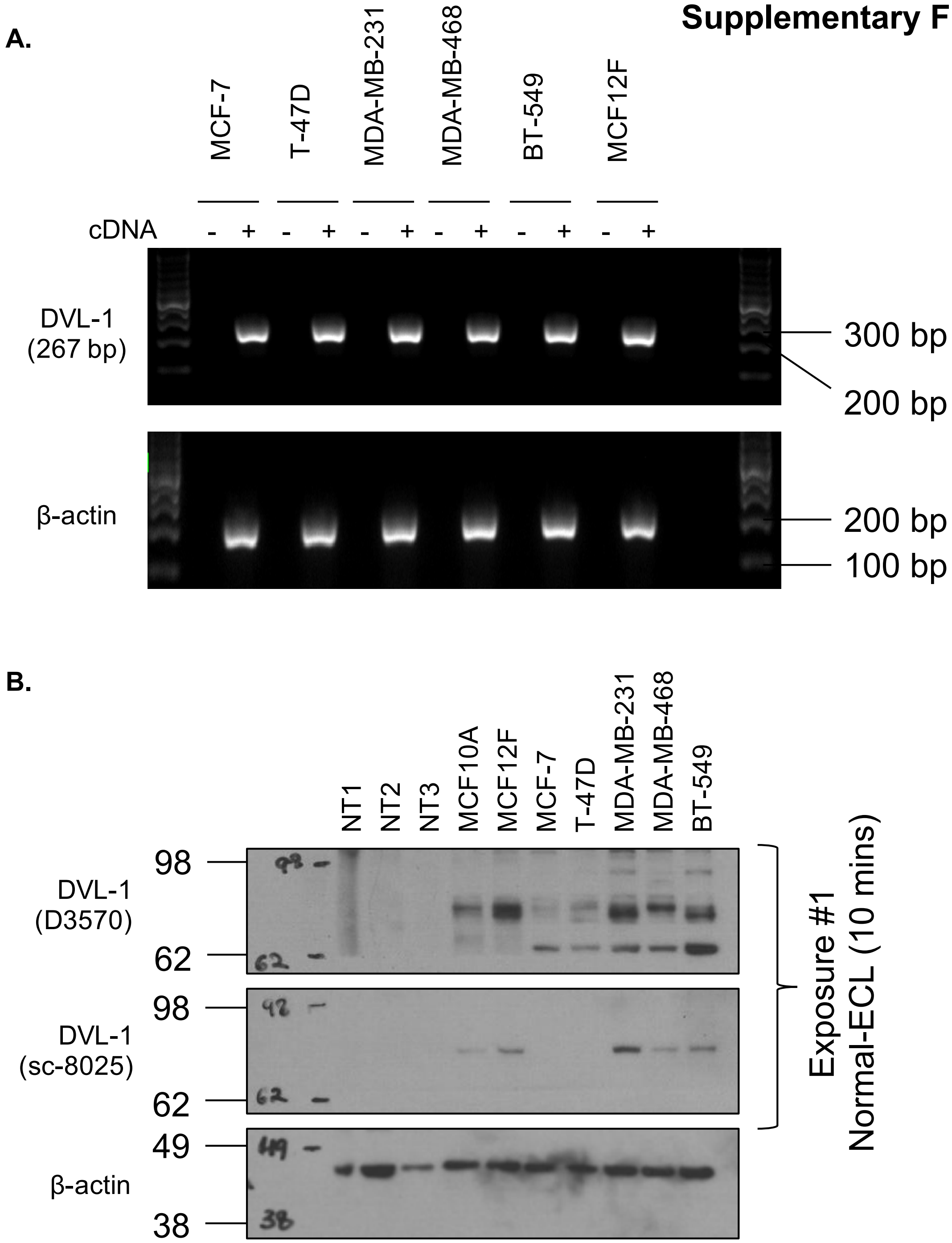

Supplementary Figure S1. Dishevelled expression in breast cancer cell lines. (A) Total mRNA was isolated from different breast cancer cell lines (MCF-7, T-47D, MDA-MB-231, BT-549 and MDA-MB-468), and a human non-cancer breast epithelial cell line (MCF12F). End-point PCR (RT-PCR) analysis of endogenous DVL-1 gene was performed using intron-spanning primers. For each amplification, either the cDNA was excluded (-) or included (+) in the reverse-transcription reaction and β-actin was included as a control. **(B)** This figure represents full-length blots for Fig. 1B with exposure times using Normal-ECL. The protein expression patterns of endogenous DVL-1 were analysed by Western blotting in breast normal whole tissue lysates (NT1, NT2, and NT3), a human non-cancer breast epithelial cell line (MCF10A and MCF12F), and different breast cancer cell lines (as described above). The membranes were probed with two different DVL-1 specific antibodies (D3570; Sigma and sc-8025; Santa Cruz Biotechnology, Inc), and β-actin was included as a control.

Supplementary Figure S2

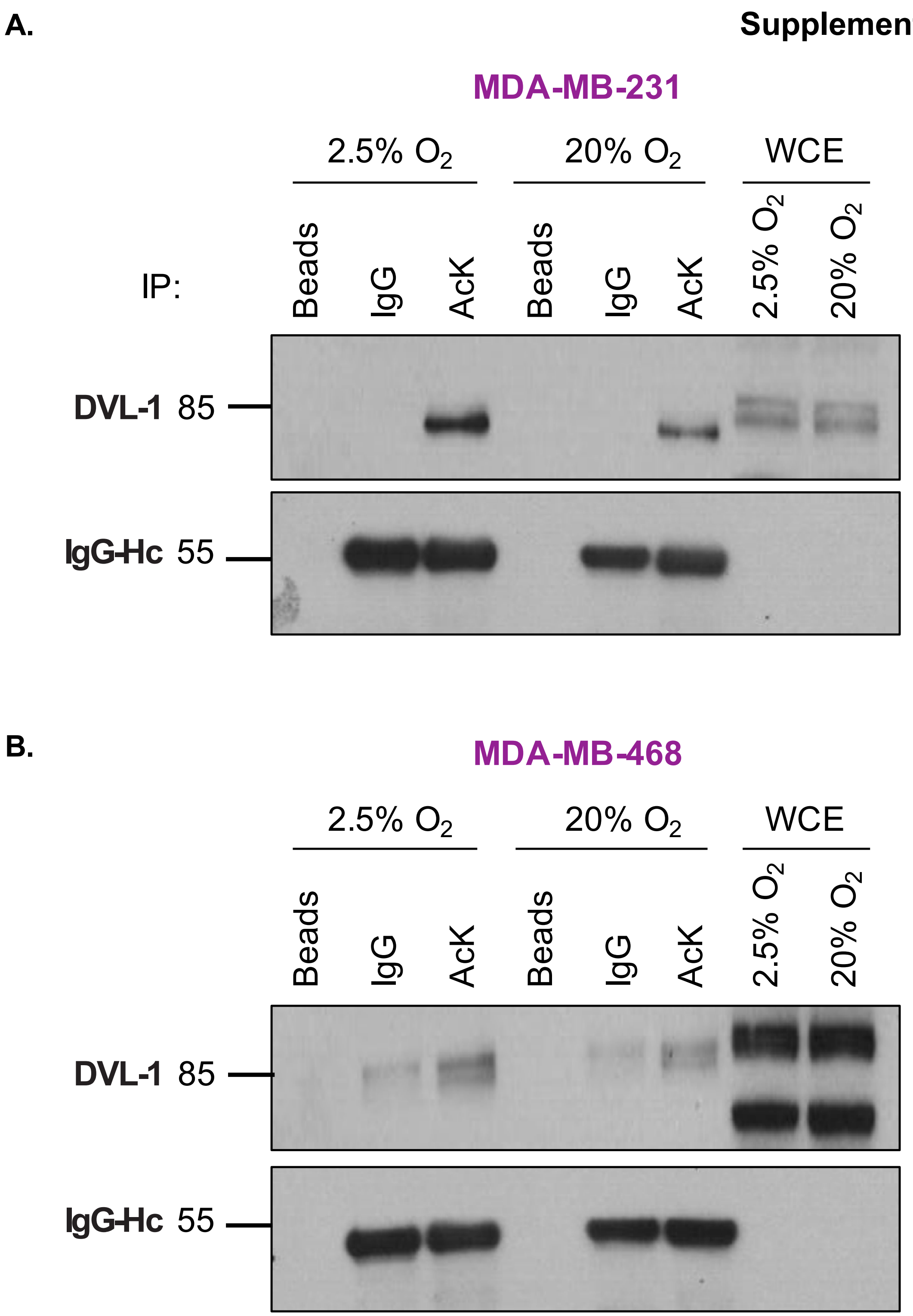

Supplementary Figure S2. DVL-1 proteins are acetylated in triple negative breast cancer cells at lower oxygen tension. MDA-MB-231 and MDA-MB-468 were cultured in two conditions, lower (2.5%) and atmospheric (20%) oxygen conditions. Equal amount of protein (2µg) was loaded for each immunoprecipitation set up using acetyl-lysine (Ac-K) antibody as per protocol. Acetylation of DVL-1 was detected by western blotting in **(A)** MDA-MB-231 **(B)** MDA-MB-468 along with positive control, whole cell extract (WCE) using DVL-1 specific antibody. Species-matched IgG and beads only were used as a negative control. IgG heavy chain (IgG Hc) was blotted for as a control for equal antibody loading for immunoprecipitation.

Supplementary Figure S3

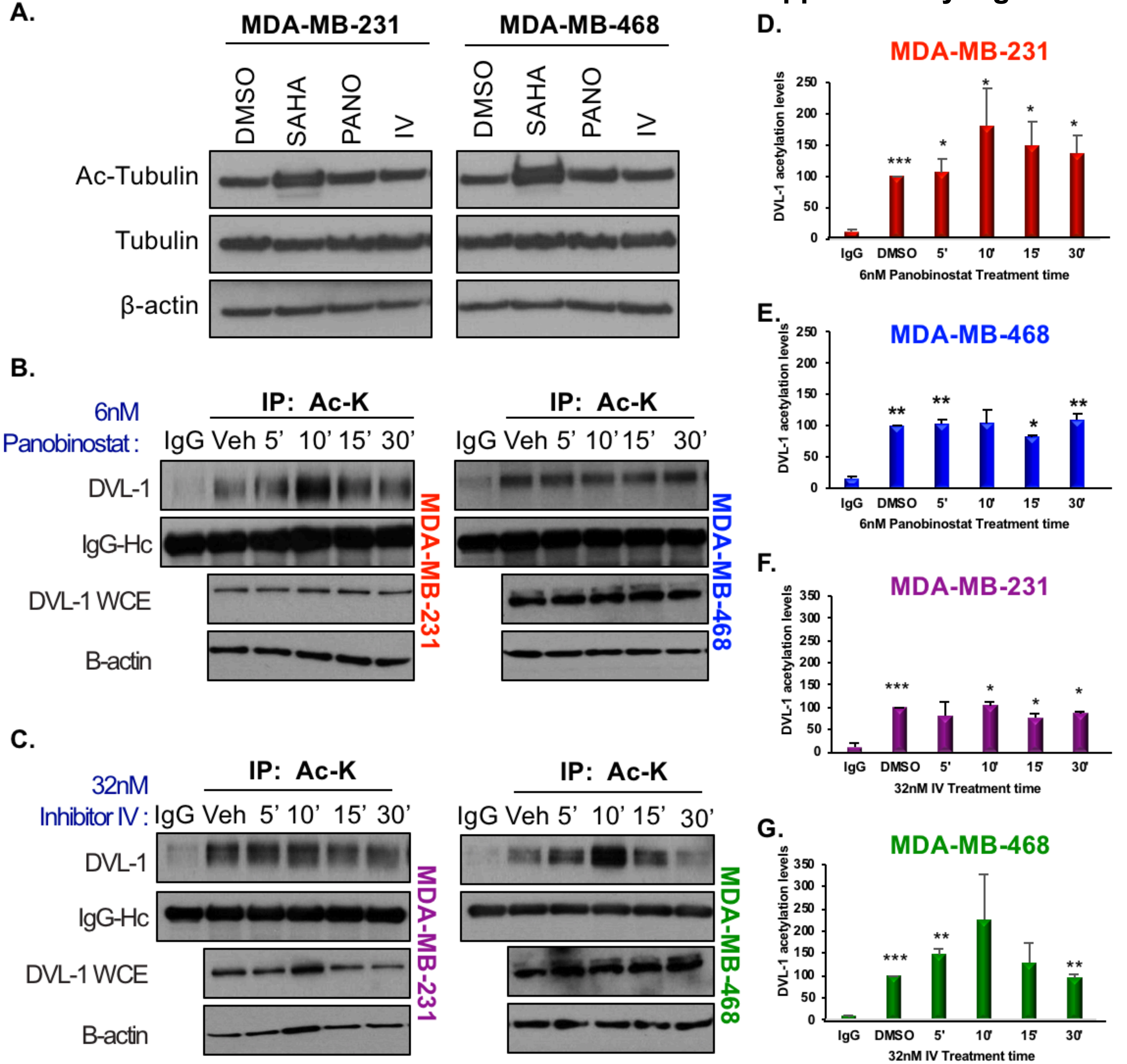

Supplementary Figure S3. Time Kinetics of DVL-1 acetylation upon deacetylase inhibition. (A) MDA-MB-231 and MDA-MB-468 were treated with deacetylase inhibitors: DMSO as vehicle control, SAHA, Panobinostat, and Inhibitor-IV. Protein lysates were subjected to western blot and probed for tubulin vs Ac-Tubulin as the internal controls for any alteration of the inhibition. 231 and 468 cells were treated with **(B)** 6nM Panobinostat and **(C)** 32nM Inhibitor-IV for a short time period range from 5 mins to 30 mins. Equal amount of protein were loaded for each immunoprecipitation set up using acetyl-lysine (Ac-K) antibody as per protocol. Acetylation of DVL-1 was detected by western blotting along with positive control, whole cell extract (WCE) using DVL-1 specific antibody. Species-matched IgG and beads only were used as a negative control. IgG heavy chain (IgG Hc) was blotted for as a control for equal antibody loading for immunoprecipitation. The graph represents relative levels of DVL-1 acetylation over short treatment time in **(D)** 231 cells treated with 6nM Panobinostat, **(E)** 468 cells treated with 6nM Panobinostat, **(F)** 231 cells treated with 32nM Inhibitor-IV, and **(G)** 468 cells treated with 32nM Inhibitor-IV. All results are expressed as mean ± SEM and considered significant at * $p<0.05$, ** $p<0.01$ and *** $p<0.001$.

Supplementary Figure S4

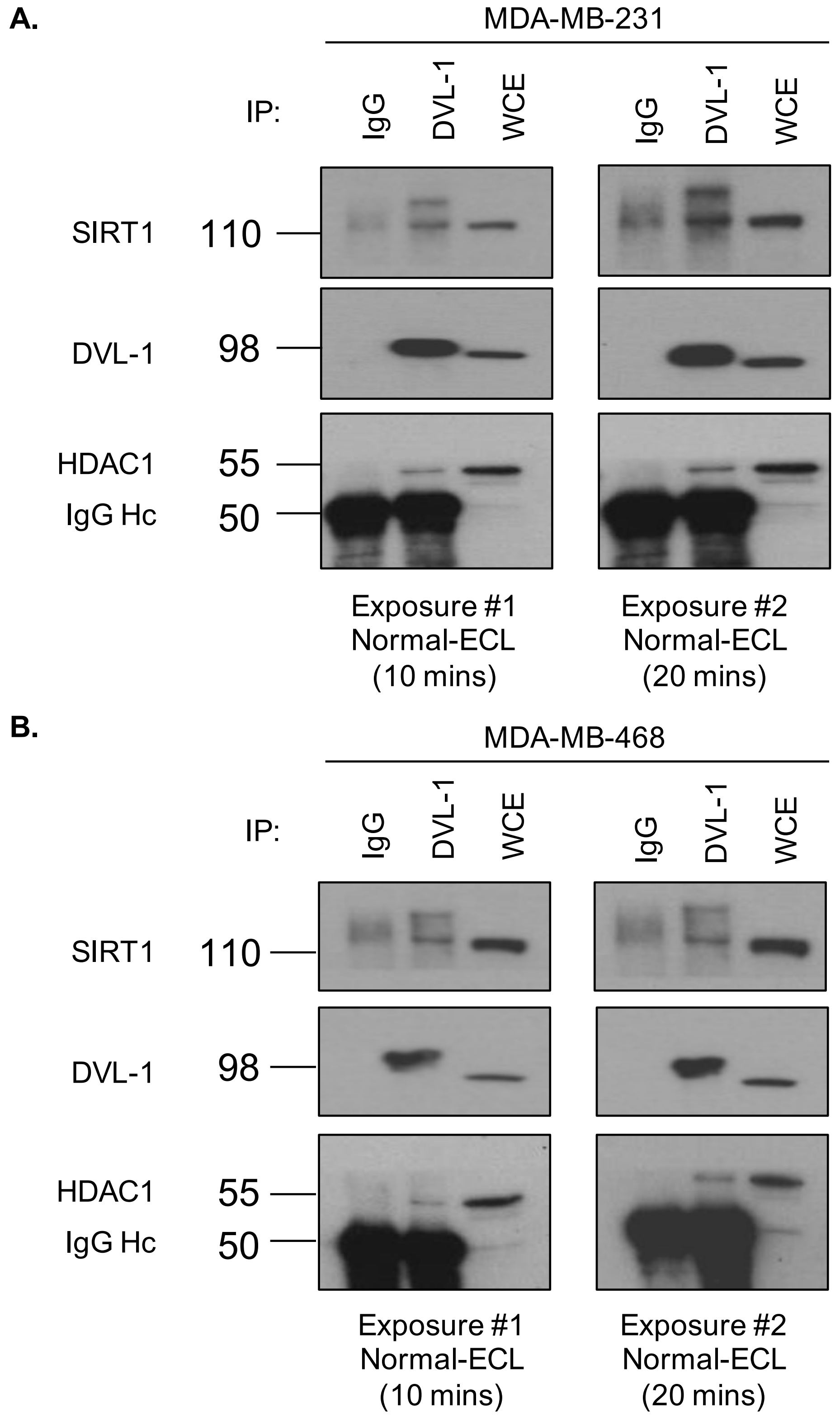

Supplementary Figure S4. Interaction between different class of deacetylases and DVL-1 proteins in multiple cell lines. This figure represents blots for Fig. 3A and Fig. 3B with different exposure time in **(A)** MDA-MB-231 and **(B)** MDA-MB-468 cells using Normal-ECL. Untreated cells were used to extract whole cell lysate for immunoprecipitation of endogenous DVL-1 with specific antibody and species-matched IgG was used as a negative control. Western blotting was performed for SIRT1 and HDAC1 from immunoprecipitated samples along with whole cell extracts (WCE).

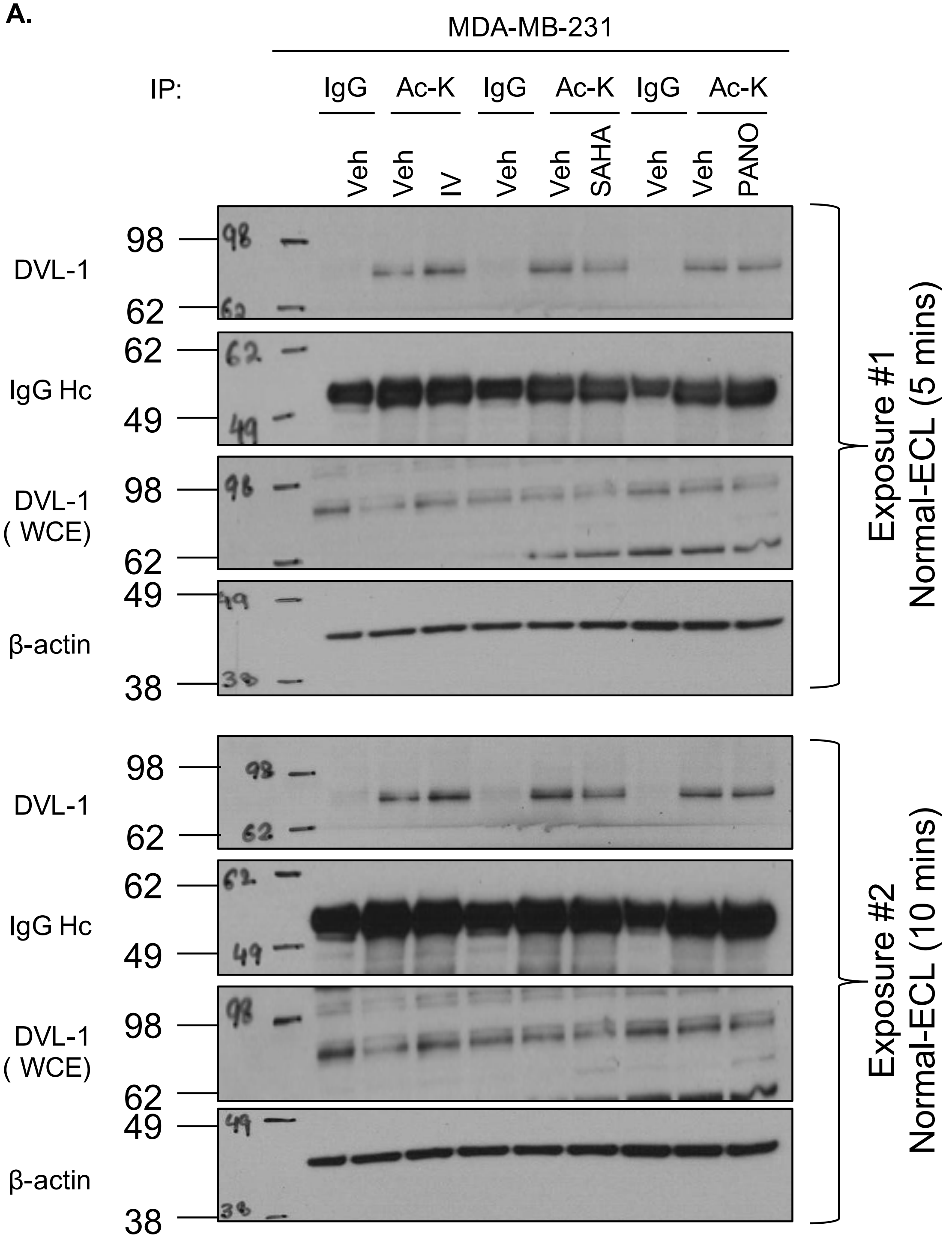

Supplementary Figure S5. Deacetylase inhibition alters DVL-1 acetylation levels in MDA-MB-231 cell lines. This figure represents full-length blots for Fig. 3C with different exposure times using Normal-ECL in MDA-MB-231 cells. Cells were treated with either DMSO (vehicle control; Veh) or 32nM inhibitor-IV (IV)**,** or 1μM SAHA**,** or 6nM Panobinostat (PANO) for 10 minutes. Equal amount of protein (2μg) was loaded for each immunoprecipitation set up using acetyl-lysine (Ac-K) antibody as per protocol. Acetylation of DVL-1 was detected by western blotting along with positive control, whole cell extract (WCE) using DVL-1 specific antibody. Species-matched IgG was used as a negative control. IgG heavy chain (IgG Hc) was blotted for as a control for equal antibody loading for immunoprecipitation. β-actin was included as a loading control for WCE.

Supplementary Figure S6

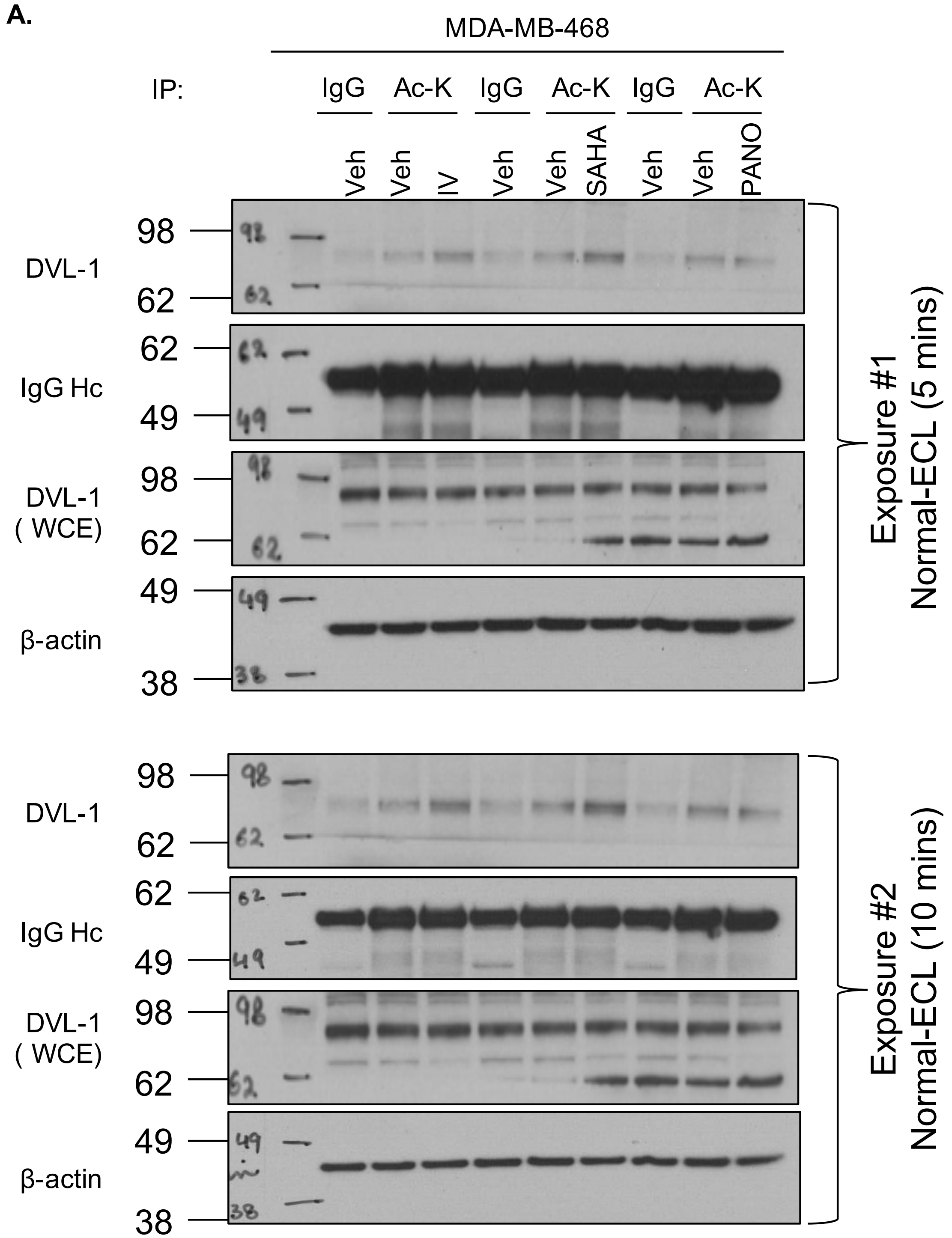

Supplementary Figure S6. Deacetylase inhibition alters DVL-1 acetylation levels in MDA-MB-468 cell lines. This figure represents full-length blots for Fig. 3D with different exposure times using Normal-ECL in MDA-MB-468 cells. Cells were treated with either DMSO (vehicle control; Veh) or 32nM inhibitor-IV (IV), or 1μM SAHA, or 6nM Panobinostat (PANO) for 10 minutes. Equal amount of protein (2μg) was loaded for each immunoprecipitation set up using acetyl-lysine (Ac-K) antibody as per protocol. Acetylation of DVL-1 was detected by western blotting along with positive control, whole cell extract (WCE) using DVL-1 specific antibody. Species-matched IgG was used as a negative control. IgG heavy chain (IgG Hc) was blotted for as a control for equal antibody loading for immunoprecipitation. β-actin was included as a loading control for WCE.

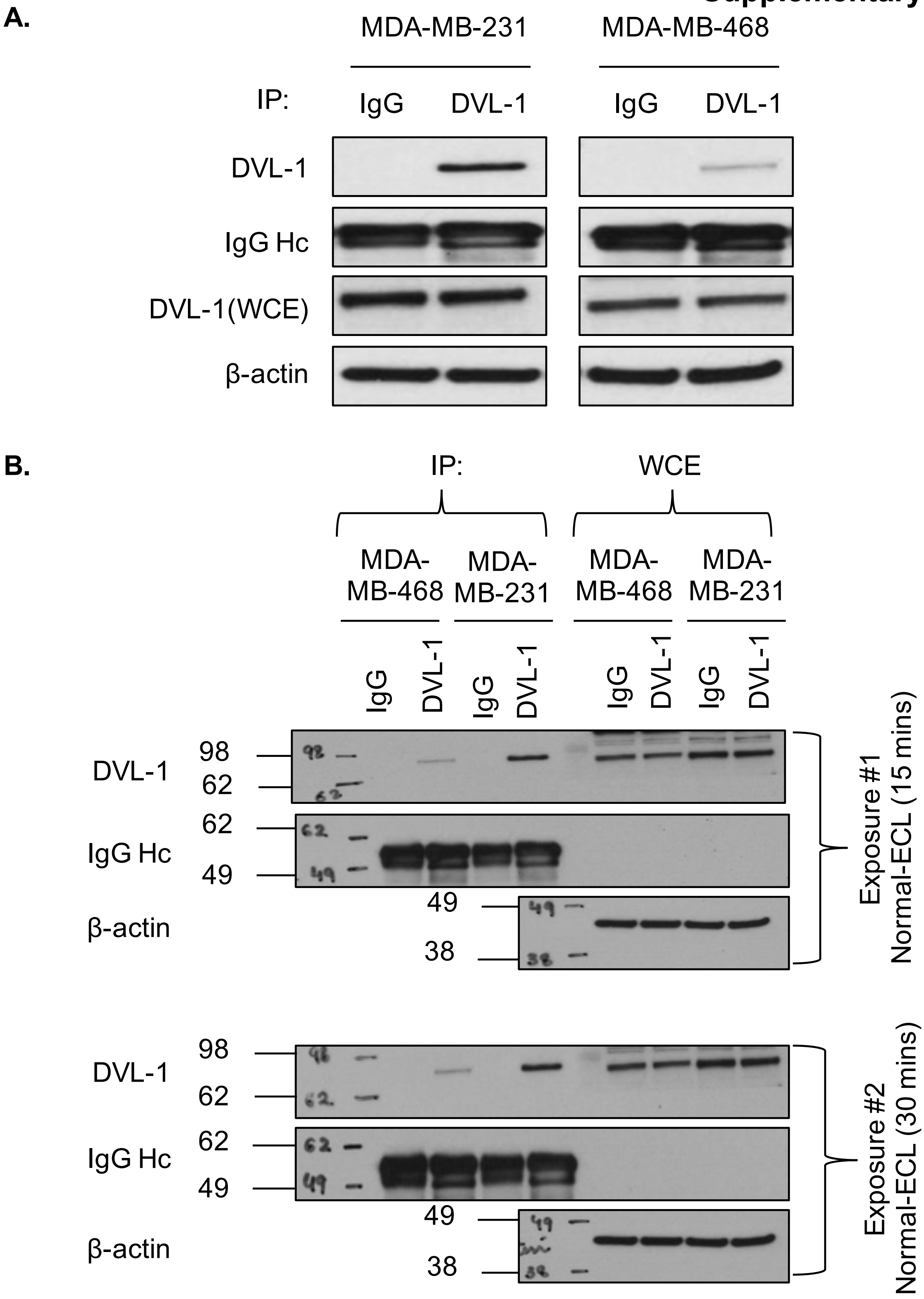

Supplementary Figure S7. Immunoprecipitation of DVL-1 proteins. (A) To determine the specificity of DVL-1 antibody, immunoprecipitation was performed for DVL-1 (D3570; Sigma) or species-matched IgG control. Western blot was performed for DVL-1 from immunoprecipitated samples along with whole cell extracts (WCE). IgG heavy chain (IgG Hc) was blotted for as a control for equal antibody loading for immunoprecipitation. β-actin was included as a loading control for WCE. **(B)** This figure represents full-length blots for Fig. S7(A) with different exposure times using Normal-ECL.

A.

Acetylated lysines on endogenous DVL-1 in TNBC cells	
K-Ac	Representative peptide
K5	AETKIIYHMDEEETPYLVK
K34	VTLADFK
K60	SMDQDFGVVK
K69	EEIFDDNAK
K285	GDGGIYIGSIMK
K476	KYASSLLK

B.

Acetylated lysines on endogenous DVL-1 in MCF10A cells	
K-Ac	Representative peptide
K34	VTLADFK
K69	EEIFDDNAK
K438	DRMWLK
K476	YASSLLKHGFLR

Supplementary Figure S8. Putative lysine residues acetylated on endogenous DVL-1. The table indicates putative lysine residues on endogenous DVL-1 that were found to be acetylated **(A)** under basal and KDI-induced conditions in MDA-MB-231 and MDA-MB-468 cells, and **(B)** in non-cancer mammary epithelial MCF10A cells, along with their representative peptide as detected by liquid chromatography mass spectrometry (LC-MS/MS) analyses.

Supplementary Figure S9

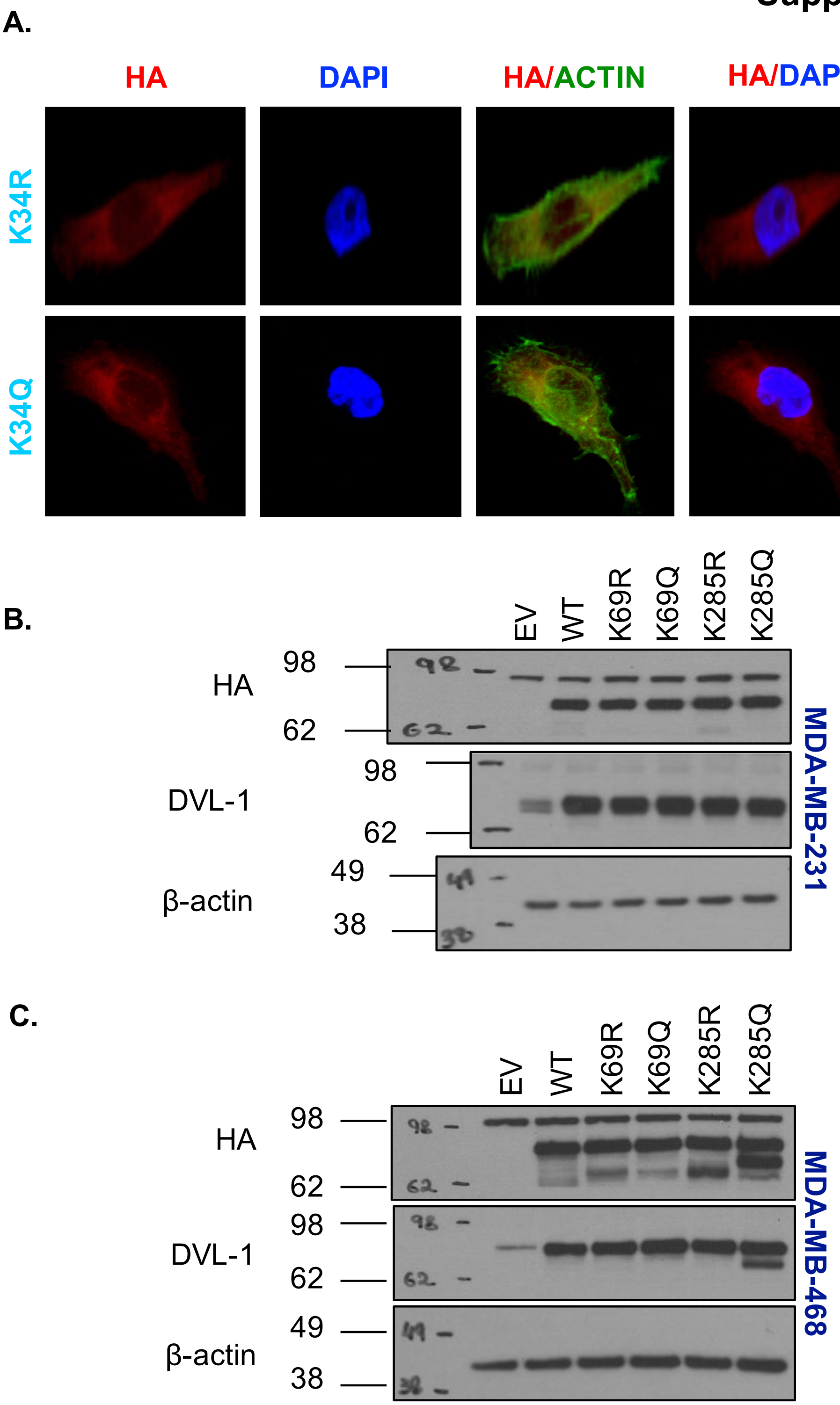

Supplementary Figure S9. Acetylation on K34 residue does not influence DVL-1 subcellular localization in MDA-MB-231 cells. (A) Immunofluorescence staining of K34R mutant (N-HA-K34R), K34Q mutant (N-HA-K34Q) proteins in stable expressing MDA-MB-231 cells. Merge of N-HA-DVL-1 (red) and nuclear staining (blue) proteins is shown as HA/DAPI for each of the mutant. Merge of actin (green) and N-HA-DVL-1 (red) proteins is shown as HA/Actin for each of the mutant. Full length images of Fig. 6A and Fig. 7A representing stable expression of empty vector (EV), N-terminal HA-epitope tagged DVL-1 wild type (WT), HA-tagged deacetylation mutants (K to R), and HA-tagged acetylation mutants (K to Q) on two lysine residues, K69 and K285 in **(B)** MDA-MB-231 and **(C)** MDA-MB-468 cells.

A.

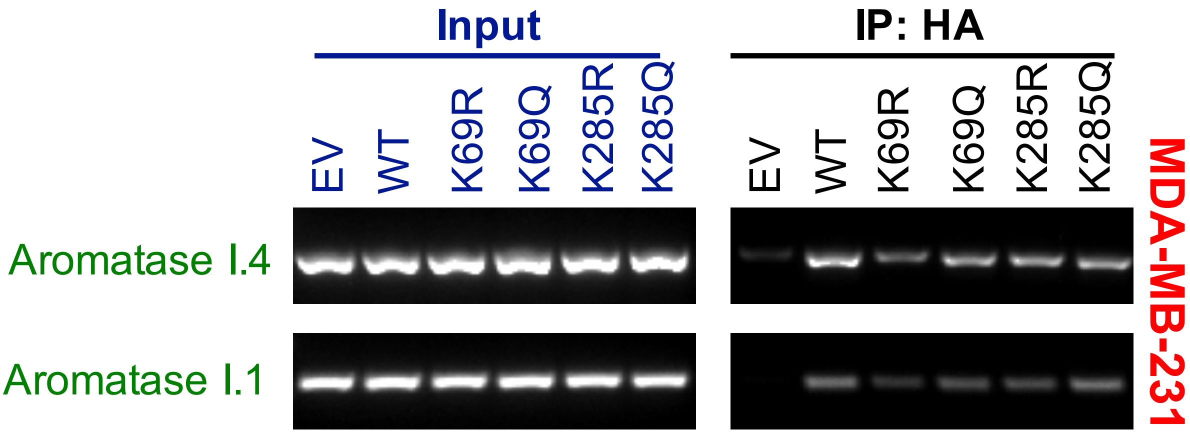

B.

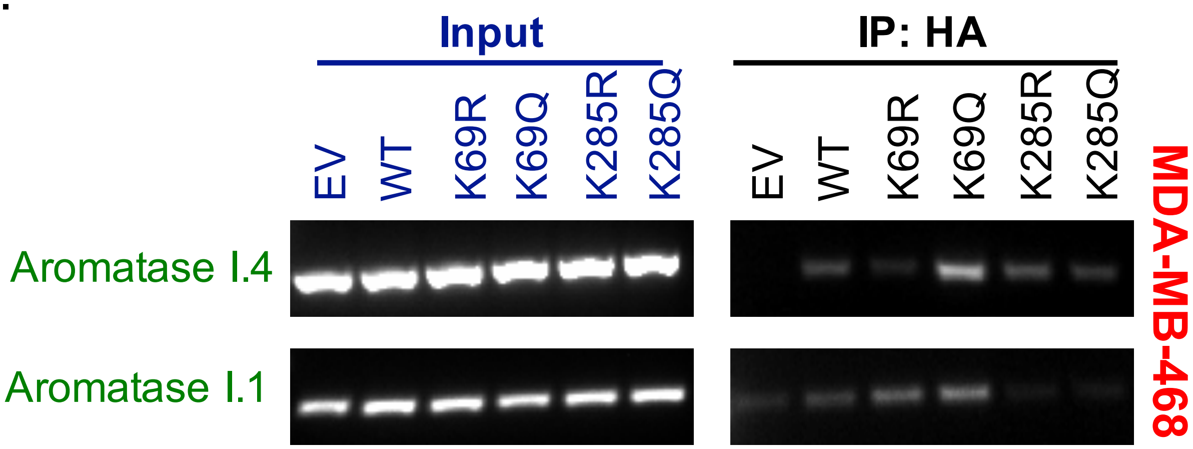

Supplementary Figure S10. DVL-1 acetylation on K69 and K285 lysine residues influences its binding to *CYP19A1* (aromatase) promoters. Occupancy of HA-tagged DVL-1 at tissue-specific promoters of *CYP19A1* gene (adipose I.4, and placental I.1 promoter) were analysed by end-point PCR. ChIP experiments for HA-tag were performed in **(A)** MDA-MB-231 and **(B)** MDA-MB-468 cells stably expressing EV (empty vector; negative control), HA-tagged wild-type DVL-1 (WT), HA-tagged deacetylation mutants (K69R and K285R), and HA-tagged acetylation mutants (K69Q and K285Q).
